# Functional Analysis of Non-Genetic Resistance to Platinum in Epithelial Ovarian Cancer Reveals a Role for the MBD3-NuRD Complex in Resistance Development

**DOI:** 10.3390/cancers13153801

**Published:** 2021-07-28

**Authors:** Tabea L. Bauer, Katrin Collmar, Till Kaltofen, Ann-Katrin Loeffler, Lorena Decker, Jan Mueller, Sabine Pinter, Stephan A. Eisler, Sven Mahner, Patricia Fraungruber, Stefan Kommoss, Annette Staebler, Lewis Francis, R. Steven Conlan, Johannes Zuber, Udo Jeschke, Fabian Trillsch, Philipp Rathert

**Affiliations:** 1 Department of Biochemistry, Institute of Biochemistry and Technical Biochemistry, University of Stuttgart, 70569 Stuttgart, Germany; tabea.bauer@ibtb.uni-stuttgart.de (T.L.B.); katrin.collmar@googlemail.com (K.C.); st148383@stud.uni-stuttgart.de (A.-K.L.); st144027@stud.uni-stuttgart.de (L.D.); st142473@stud.uni-stuttgart.de (J.M.); sabine.pinter@ibtb.uni-stuttgart.de (S.P.); 2 Department of Obstetrics and Gynecology, University Hospital, LMU Munich, 81377 Munich, Germany; till.kaltofen@med.uni-muenchen.de (T.K.); Sven.Mahner@med.uni-muenchen.de (S.M.); P.Fraungruber@campus.lmu.de (P.F.); Udo.Jeschke@med.uni-muenchen.de (U.J.); 3 Stuttgart Research Center Systems Biology (SRCSB), University of Stuttgart, 70569 Stuttgart, Germany; stephan.eisler@srcsb.uni-stuttgart.de; 4 Department of Women’s Health, Tubingen University Hospital, 72076 Tuebingen, Germany; stefan.kommoss@med.uni-tuebingen.de; 5 Institute of Pathology and Neuropathology, Tuebingen University Hospital, 72076 Tuebingen, Germany; annette.staebler@med.uni-tuebingen.de; 6 Swansea University Medical School, Singleton Park, Swansea SA2 8PP, UK; l.francis@swansea.ac.uk (L.F.); R.S.Conlan@swansea.ac.uk (R.S.C.); 7 Research Institute of Molecular Pathology, Vienna BioCenter, 1030 Vienna, Austria; johannes.zuber@imp.ac.at; 8 Medical University of Vienna, Vienna BioCenter (VBC), 1030 Vienna, Austria; 9 Department of Obstetrics and Gynecology, University Hospital Augsburg, 86156 Augsburg, Germany

**Keywords:** non-genetic platinum resistance, MBD3, epithelial ovarian cancer, enhancer remodeling, biomarker, RNAi screen

## Abstract

**Simple Summary:**

Most epithelial ovarian cancer (EOC) patients, although initially responsive to standard treatment with platinum-based chemotherapy, develop platinum resistance over the clinical course and succumb due to drug-resistant metastases. It has long been hypothesized that resistance to platinum develops as a result of epigenetic changes within tumor cells evolving over time. In this study, we investigated epigenomic changes in EOC patient samples, as well as in cell lines, and showed that profound changes at enhancers result in a platinum-resistant phenotype. Through correlation of the epigenomic alterations with changes in the transcriptome, we could identify potential novel prognostic biomarkers for early patient stratification. Furthermore, we applied a combinatorial RNAi screening approach to identify suitable targets that prevent the enhancer remodeling process. Our results advance the molecular understanding of epigenetic mechanisms in EOC and therapy resistance, which will be essential for the further exploration of epigenetic drug targets and combinatorial treatment regimes.

**Abstract:**

Epithelial ovarian cancer (EOC) is the most lethal disease of the female reproductive tract, and although most patients respond to the initial treatment with platinum (cPt)-based compounds, relapse is very common. We investigated the role of epigenetic changes in cPt-sensitive and -resistant EOC cell lines and found distinct differences in their enhancer landscape. Clinical data revealed that two genes (JAK1 and FGF10), which gained large enhancer clusters in resistant EOC cell lines, could provide novel biomarkers for early patient stratification with statistical independence for JAK1. To modulate the enhancer remodeling process and prevent the acquisition of cPt resistance in EOC cells, we performed a chromatin-focused RNAi screen in the presence of cPt. We identified subunits of the Nucleosome Remodeling and Deacetylase (NuRD) complex as critical factors sensitizing the EOC cell line A2780 to platinum treatment. Suppression of the Methyl-CpG Binding Domain Protein 3 (MBD3) sensitized cells and prevented the establishment of resistance under prolonged cPt exposure through alterations of H3K27ac at enhancer regions, which are differentially regulated in cPt-resistant cells, leading to a less aggressive phenotype. Our work establishes JAK1 as an independent prognostic marker and the NuRD complex as a potential target for combinational therapy.

## 1. Introduction

Epithelial ovarian cancer (EOC) is the most lethal disease of the female reproductive tract and associated with high morbidity and mortality rates. Commonly diagnosed in advanced stages, the incidence of EOC in 2020 was projected to be 314,000 new cases and 207,000 deaths worldwide [1]. Standard treatment for advanced EOC consists of radical surgery, including multivisceral procedures to achieve complete cytoreduction, followed by platinum- and taxane-based combination chemotherapy. Since 2011, systemic treatment is usually expanded by bevacizumab and recently poly ADP-ribose polymerase (PARP) inhibitors [2,3]. Although most patients respond to this multimodal treatment, relapses are common and patients with advanced EOC have a median overall survival of ~65 months [4]. As the overall survival (OS) for EOC patients has not significantly improved over the past 20 years, there is an urgent need for new strategies that improve EOC treatment and prognosis [5].

The overall rate of somatic mutations in platinum-resistant and -sensitive EOC tumors is highly comparable [6] and acquired resistance to platinum-based therapy has been associated with non-genetic transient molecular epigenetic mechanisms in EOC [7], similar to other tumors [8,9], conferring advantageous cellular plasticity in complex tumor environments [10,11]. Recent papers have highlighted a role of enhancers in EOC [12], resulting in the dysregulation of major regulatory signaling pathways [13,14] in EOC.

Many epigenetic co-regulator complexes play an important role in cancer biology and resistance development [15], among them the nucleosome remodeling and histone deacetylase (NuRD) complex. The NuRD complex was shown to regulate oncogenesis and cancer progression decades ago [16] and to bind to active promotors and enhancers in several cell types, regulating their accessibility [17]. The NuRD complex is formed by different subunits and can elicit different functions depending on the combination of subunits [18]. The methyl CpG binding domain 3 (MBD3) is a core subunit of NuRD, which maintains its structural integrity [17].

In the present study, we analyzed histone H3 lysine 27 acetylation (H3K27ac), a mark of active enhancers, in paired cPt-sensitive and -resistant EOC cell lines and could show that platinum resistance is accompanied by profound remodeling of the enhancer landscape. These differential enhancers lead to the upregulation of genes, including *JAK1* and *FGF10* in resistant cells, which can serve as independent biomarkers predictive for overall survival in EOC patients. To further characterize and explore means to interfere with the process of acquired non-genetic platinum resistance, we performed a chromatin-focused RNAi screen in a cPt-sensitive EOC cell line treated with cPt. Our differential top hits, which showed strong depletion in the presence of cPt, comprised components of the NuRD complex, including MBD3. Suppression of MBD3 expression prevented the development of cPt resistance and induced distinct alterations in H3K27ac, leading to an inverse effect on differential enhancer regions that were identified in A2780cis cells and previously linked to resistance mechanisms.

## 2. Materials and Methods

Extended information on experimental procedure is available in the Supplementary Materials’ Material and Methods.

### 2.1. Plasmids

The shRNA library was cloned into the SGEN vector (pRRL-SFFV-GFP-miRE-PGK-NeoR). Validation of the hits was performed by using the LT3GEN vector (pRRL-TRE3G-GFP-miRE-PGK-NeoR) [19]. Guide sequences of the shRNAs are described (Table S1).

### 2.2. Antibodies

Immunofluorescence staining: anti-γH2A.X (Cell Signaling Technology, 2577), anti-Rad51 (Abcam, ab133534) and goat anti-Rabbit IgG secondary antibody, Alexa Fluor 594 (Invitrogen, A11037). Western blot: anti-MBD3 antibody (Abcam, ab91458), anti-Histone H3 antibody (Abcam, ab24834), anti-ß Actin antibody (Abcam, ab8227), IRDye^®^ 800CW Donkey anti-Rabbit IgG secondary antibody (Licor, 926-32213) and IRDye^®^ 680RD Donkey anti-Mouse IgG secondary antibody (Licor, 926-68072). ChIP: anti-Histone H3 (acetyl K27) antibody (Abcam, ab4729), anti-Histone H3 (acetyl K9) antibody (Abcam, ab12179), normal Rabbit IgG Control (R&D, AB-105-C) or anti-mouse IgG (Molecular Probes, 6691-1). Immunohistochemistry: anti-Histone H3 (acetyl K27) antibody (Abcam, ab177178), anti-Histone H3 (acetyl K9) antibody (Abcam, ab32129), anti-FGF10 antibody (Genetex, GTX12469) and anti-JAK1 (Cell Signaling Technology, 3344).

### 2.3. Experiments on Human Tissue Samples

Formalin-fixed and paraffin-embedded (FFPE) tissue specimens of 365 patients with a first diagnosis of EOC undergoing primary radical cytoreductive surgery were analyzed. Clinical data were collected, and information about the follow-up was acquired. Staging and grading were performed by following the TNM and FIGO (International Federation of Gynecology and Obstetrics) classification. A summary of patient characteristics is provided (Table S2). Sampling, microarray construction and immunohistochemical staining were performed as previously described [20]. Evaluation, imaging and storing were performed with an AxioScope microscope (Carl Zeiss, Jena, Germany); the immune-reactivity score (IRS) was assessed semi-quantitatively by two examiners, and mean values of the three representative IHC scores of every probe were calculated.

### 2.4. Cell Culture, Generation of Tet-On Competent Cells and Lentiviral Transduction

Cell lines were purchased from Sigma-Aldrich supplied by ECACC or ATTC and authenticated. CAOV4cis cells were generated by prolonged treatment of CAOV4 cells for 21 days with increasing concentrations of cPt. All media were supplemented with 10% Fetal Bovine Serum, 4 mM L-Glutamine, 10 mM HEPES, 1 mM Sodium pyruvate solution, 100 U/mL Penicillin and 100 µg/mL Streptomycin. A2780, A2780cis, CAOV4 and CAOV4cis cells were cultured in supplemented RPMI 1640, and Lenti-X 293T cells were cultured in supplemented DMEM. Media for the resistant A2780cis and CAOV4cis cells were additionally supplemented with 1 µM cPt. Generation of Tet-on competent cells was performed as previously described [21]. LT3GEN vectors for expression of shRNAs were introduced into the Tet-on competent target cells by lentiviral transduction and at a transduction efficiency <20% to ensure single plasmid integration. For selection, cells were treated with 1–3 mg/mL G418 solution, and knockdown was induced by the addition of 1 µg/mL Doxycycline (Dox).

### 2.5. Viability Assay for cPt GI_50_ Determination

96-well plates were seeded with 2500 cells/well for CAOV4 and 1 × 10^4^ cells/well for A2780, A2780cis and CAOV4cis. Cells were treated with increasing concentrations of 0–81 µM cPt for 72 h. Cell viability was determined by CellTiter-Glo^®^ Luminescent Cell Viability Assay (Sigma-Aldrich, Taufkirchen, Germany) according to the manufacturer’s instructions.

### 2.6. MBD3 Protein Expression Western Blot

Western blots to confirm MBD3 knockdown were performed as previously described [22]. In brief, cells expressing shRNAs for 7 days were acquired by cell sorting, lysed and proteins resolved by SDS–PAGE. Blotting was performed by using a wet-tank blotting system (BioRad, Hercules, CA, USA), and membranes were stained with primary antibodies anti-MBD3 (1:5000), anti-histone H3 (1:2000) or anti-β-actin (1:5000). Membranes were stained with secondary antibodies IRDye^®^ 800CW (LI-COR Biosciences, Lincoln, NE, USA) or IRDye^®^ 680RD (LI-COR Biosciences, Lincoln, NE, USA) (1:15,000) and imaged by using the Odyssey Infrared Image System (LI-COR Biosciences, Lincoln, NE, USA).

### 2.7. Multiplexed RNAi Screen

The multiplexed RNAi screen was performed as previously described [21]. In brief, an shRNA library targeting 1139 chromatin associated genes (6485 shRNAs) was transduced into A2780 cells. After 7 days of selection, cells were treated with vehicle or 1 µM cPt for an additional 7 days. GFP+ cells were acquired by cell sorting, and genes synergizing with cPt were identified by deep-sequencing of the initial library pool and the remaining shRNA guides after 14 days of culture.

### 2.8. Immunofluorescence Staining, Image Acquisition and Analysis

Immunofluorescence staining and image acquisition were performed as previously described [22]. In brief, cells expressing shRNAs for 7 days were treated with 1 µM cPt or vehicle for 24 h. Cells were fixed, permeabilized and, after blocking, stained with primary antibody anti-γH2A.X (1:500) or anti-Rad51 (1:1000). Cells were subsequently stained with secondary antibody Alexa Fluor 594 (1:1000) and cell nuclei using 1 µg/mL of DAPI. Slides were mounted onto microscopy slides, using Mowiol^®^ 4–88 (Sigma-Aldrich, Taufkirchen, Germany) and images acquired by using a Zeiss Cell Observer Z1 epifluorescence microscope (Carl Zeiss, Jena, Germany). Quantitative analysis was performed by CellProfiler (https://cellprofiler.org/; accessed on 26 July 2021) version 3.1.8 [23].

### 2.9. Gene-Expression Analysis 

Cells were harvested following cPt treatment for the indicated time points, and mRNA was extracted by using the RNeasy Plus Mini Kit (QIAGEN, Hilden, Germany). Then mRNA was transcribed into cDNA, and gene expression was determined by real-time quantitative PCR (RT-qPCR). Primers for RT-qPCR are described in Table S3.

### 2.10. Chromatin Immunoprecipitation (ChIP-qPCR and ChIP-Seq) 

ChIP was performed as previously described [24]. In brief, 2.5 × 10^6^ cells were harvested and washed with PBS supplemented with 500 nM Trichostatin A. For ChIP-seq of MBD3 knockdown cells, cells were selected for 7 days and knockdown induced for an additional 7 days. Then 5 × 10^6^ GFP-positive cells for each shRNA were acquired by FACS and washed with PBS supplemented with 500 nM Trichostatin A. Mononucleosomes were prepared and supplemented with spike-in control (5% of total, mononucleosomes, *D. melanogaster*). ChIPs were performed with 2.5 µg of respective antibody, and ChIP-qPCR samples were quantified by using the CFX Real-Time PCR detection system (Bio-Rad, Hercules, CA, USA) and normalized to the input and the spike-in control. Primers used for ChIP-qPCR are described in Table S4.

### 2.11. Analysis of ChIP-Seq Data 

Sequencing was performed on an Illumina HiSeq 3000 deep sequencer, acquiring 2 × 150 bp paired end reads. Sequencing data were analyzed, using the galaxy platform (https://usegalaxy.eu/, accessed on 26 July 2021) [25]. In brief, reads were aligned, using Bowtie2 [26]. The ChIP-seq data were quantile normalized, using a custom-made R script (https://github.com/Rathert-lab, accessed on 26 July 2021), and peaks were called by using MACS2 (accessed on 26 July 2021) [27]. Differential peak filtering was performed by selecting peaks >2 kb, average peak intensity >1000 and a fold change between upregulated and downregulated regions >1.3 or <0.75, respectively. Super-enhancers were called by using the Rank Ordering of Super-Enhancers (ROSE, accessed on 26 July 2021) tool [28]. Gene-set enrichment analysis was performed by using ChIP-Enrich [29]. For SNP frequency analysis, genetic variants were called from the ChIP-seq datasets, using FreeBayes (accessed on 26 July 2021) [30]. A custom-made script (https://github.com/Rathert-lab, accessed on 26 July 2021) was used to extract the number of SNPs per region from the FreeBayes output.

ChIP-seq data are available via Gene Expression Omnibus (GSE172510).

### 2.12. Analysis of RNA-Seq Data of EOC Cell Lines and Patient Samples 

RNA-seq data were obtained from a previously published dataset for A2780 and A2780cis cells (GSE98230) [31]. Raw counts of A2780 and A2780cis cells were extracted and processed on the galaxy platform (https://usegalaxy.eu/; accessed on 26 July 2021) [25].

### 2.13. Statistical Analyses 

Statistical analysis was performed in GraphPad Prism version 9.1.0 (GraphPad Software, San Diego, CA, USA). Statistics of patient data were determined by using SPSS Statistics 25 (IBM, Chicago, IL, USA).

## 3. Results

### 3.1. Non-Genetic Resistance in EOC Is Associated with Changes in the Enhancer Landscape 

**Figure 1:**
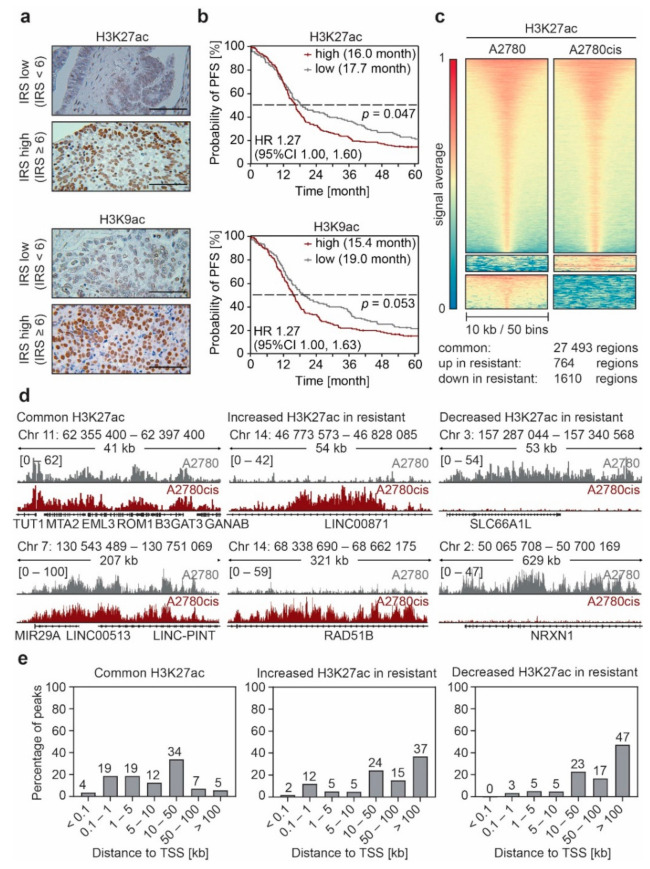
Non-genetic resistance to platinum (cPt) in epithelial ovarian cancer (EOC) is associated with changes in the enhancer landscape. (**a**) Representative immunohistochemistry (IHC) images of EOC patient tissue stained against H3K27ac or H3K9ac evaluated by the immune reactive score (IRS) to attribute staining intensity of the specimens for the Kaplan–Meier survival analysis as shown in B. Tissue samples with low (IRS < 6) or high (IRS ≥ 6) modification levels are shown (scale bar 100 μm). (**b**) Kaplan–Meier survival analysis of progression-free survival (PFS) of 365 patients with high-grade serous EOC with low or high H3K27ac or H3K9ac modification levels. Median survival of each group is indicated. Statistical significance was determined by Chi-Square statistics of the Log-Rank test (Mantel–Cox). (**c**) Heatmaps showing the average H3K27ac ChIP-seq signal for A2780 and A2780cis EOC cells. Signals are sorted by the highest average signal in A2780 cells and plotted with a 10 kb window around the peak center. Regions with differential H3K27ac signal between sensitive and resistant cells were identified by K-means clustering. (**d**) Representative ChIP-seq tracks of H3K27ac intensity in A2780 and A2780cis cells for regions with common and differential H3K27ac peaks identified in (**c**). (**e**) Distribution of the distance of the common and differential H3K27ac peaks from (**c**) to the nearest transcription start site (TSS) identified by ChIP-Enrich.

In order to investigate the epigenetic landscape in EOC, we analyzed a well characterized cohort of 365 patients with primary EOC by immunohistochemistry (IHC). We stained samples for global histone 3 lysine 9 and lysine 27 acetylation (H3K9ac, H3K27ac), which are well-documented enhancer-associated histone modifications [32], and correlated acetylation with survival (Table S2, and Figure 1a). High levels of H3K9ac and H3K27ac were associated with both decreased overall (OS) and progression-free survival (PFS) which was statistically significant for H3K27ac regarding PFS (Figure 1b) and for H3K9ac regarding OS (Figure S1). Recent literature shows that repeated treatment of EOC cell lines with cPt leads to a dysregulation in gene expression and a redistribution of enhancers [13,14]. Hence, we selected the cPt-sensitive cell line A2780 derived from an endometrioid adenocarcinoma and its cPt-resistant counterpart A2780cis, which is a well-recognized cell model for platinum resistance, for further analyses (Figure S2a). We profiled both cell lines for alterations in H3K27ac and H3K9ac by ChIP-seq. Globally we did not detect strong differences in H3K27ac or H3K9ac between the cPt-sensitive A2780 and the cPt-resistant A2780cis cells (Supplementary Figure S2b). Remarkably, we identified two distinct clusters of differentially acetylated regions in the cPt-resistant cells (Figure 1c,d; Supplementary Materials Figures S2c,d and S3). Specific regions in the cPt-resistant cell lines showed a remarkable gain or loss of H3K27ac and H3K9ac when compared to the sensitive A2780 cells. H3K9ac signals strongly correlated with H3K27ac signals in these differential regions in sensitive and resistant cells (Figure S2e). To understand whether the identified H3K27ac alterations were common to EOC, we evaluated another EOC subtype and continued the investigation with the high-grade serous EOC cell line CAOV4 and the cPt-resistant CAOV4cis (Figure S4a). Again, we identified differential H3K27ac clusters in this cell line pair with no global changes (Figure S4b–d). The observed alterations were less pronounced compared to the A2780/A2780cis cell line pair, which we attributed to the shorter cPt treatment period of the CAOV4cis cells. Interestingly, differential H3K27ac signals did not correlate between cell line pairs (Figure S4e), suggesting that different resistance mechanisms might be involved, depending on the EOC subtype. However, in both cell line pairs most differential regions are located at sites distant to the transcription start site (TSS) (Figure 1e; Figure S2f and S4f), which fitted to the initial hypothesis that enhancer remodeling drives resistance to cPt in EOC cell lines.

In order to investigate if the remodeling of the enhancer landscape is due to accumulation of single nucleotide polymorphisms (SNPs) leading to the generation of novel binding motifs for transcription factors and co-regulators, we extracted the SNP frequency of the identified common and differential regions from the H3K27ac ChIP-seq profiles and compared the SNP frequency between sensitive and resistant cells. While we were unable to detect any differences between the SNP frequencies in the common regions of A2780 and A2780cis, loci with elevated H3K27ac signal in resistant A2780cis cells showed an increase in SNPs compared to the sensitive cells (Figure S5a). However, we did not detect any differences in the CAOV4 and CAOV4cis cells, which were exposed to cPt for a shorter period (Figure S5b) suggesting that acquired SNPs are not the reason for the observed enhancer remodeling.

### 3.2. Enhancer Remodeling Is Associated with Cellular Processes Implicated in Resistance Development 

**Figure 2:**
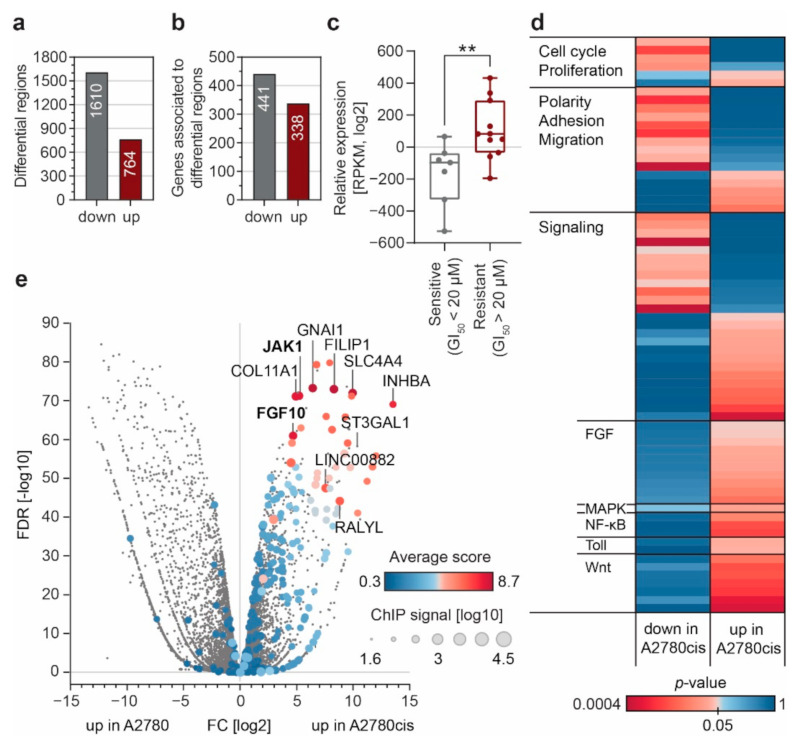
Enhancer remodeling during cPt resistance acquisition leads to gene expression changes and altered regulatory pathways. (**a**) Number of genomic regions associated with decreased (down) or increased (up) H3K27ac signal in resistant A2780cis cells as compared to sensitive A2780 cells. (**b**) Number of genes associated with the differential H3K27ac regions in (**a**), determined by ChIP-Enrich. (**c**) Gene expression of resistance associated genes between cPt-sensitive and -resistant EOC cells lines curated from an independent RNA-seq dataset of EOC cell lines (CCLE). The resistance index was plotted for all cell lines showing a GI_50_ >20 µM (resistant, *n* = 11) or a GI_50_ < 20 µM (sensitive, *n* = 7). Data are shown as a box plot with all data points displayed. Statistical significance was determined by an unpaired *t*-test (**: *p* ≤ 0.01). (**d**) Gene-set-enrichment analysis for all genes associated to regions of increased (up) or decreased (down) H3K27ac in resistant A2780cis cells. All identified pathways with a *p*-value ≤ 0.05 in either category were sorted according to the indicated categories. (**e**) Volcano plot showing gene-expression changes between sensitive A2780 and resistant A2780cis cells. Fold change in gene expression is plotted over the FDR value of the respective gene. Cumulative ChIP-seq signal intensity for each gene is indicated by dot size. Color intensity indicates average score for genes associated with increased H3K27ac in resistant cells.

We annotated the genes associated with the differential H3K27ac peaks, using ChIP-Enrich [29] (Figure 2a,b), and used curated expression data for 18 EOC cell lines of different subtypes and known GI_50_ values for cPt (Table S5) from the Cancer Cell Line Encyclopedia (CCLE) [33]. Application of a resistance score [21] significantly distinguished sensitive and resistant EOC cell lines according to their GI_50_ values (Figure 2c). A thorough analysis of associated pathways [34,35,36] revealed that many cellular processes previously connected to resistance development in other contexts are also associated with regions that gain H3K27ac in resistant A2780cis cells (Figure 2d and Table S6). We observe a clear accumulation of signaling pathways and pathways regulating key cellular processes involved in cancer biology. Moreover, we noticed a strong gain of H3K27ac signals at genes associated with the Wnt, MAPK, TLR, NF-κB and FGF signaling pathways, all of which have been connected to resistance or more aggressive disease progression in EOC [37,38,39] and other tumors [21,40]. This indicates that enhancer remodeling can facilitate cPt resistance development via upregulation of key regulatory pathways.

To identify genes which are most likely defining the sensitive or resistant phenotype, we investigated the presence of so called super-enhancers [28] in A2780 and A2780cis cells (Figure S6a). As expected, the super-enhancer associated genes could distinguish the resistance associated phenotype and super-enhancer associated genes were up or down regulated in A2780cis or A2780 respectively (Figure S6b). However, we could not detect a strong correlation between the overall expression level of genes and the H3K27ac signal associated with these genes (Figure S6b,c). This suggests that the super-enhancer status is not always connected to high gene expression, which is most likely due to the limitation of bioinformatics tools to assign enhancers to specific genes [41,42,43]. Therefore, we ranked all genes associated with increased H3K27ac regions in resistant cells and devised a score considering the fold change of expression, the reproducibility among the replicates (*p*-value) and the cumulative H3K27ac ChIP-seq signal. Interestingly, we found the genes *JAK1* and *FGF10*, associated with MAPK and FGF signaling, among the top ten most differentially expressed genes (Figure 2e). The JAK/STAT pathway is one of the major signaling pathways that is aberrantly activated in EOC and targeting of JAK1 by small molecule inhibitors has been suggested previously [44]. Similarly, FGF signaling leads to the activation of resistance associated intracellular signaling pathways, including the STAT, PI3K and MAPK pathways, and has been implicated in cPt resistance [39,45].

### 3.3. FGF10 and JAK1 Expression Is Associated with Impaired Prognosis and JAK1 Is an Independent Predictive Biomarker 

**Figure 3:**
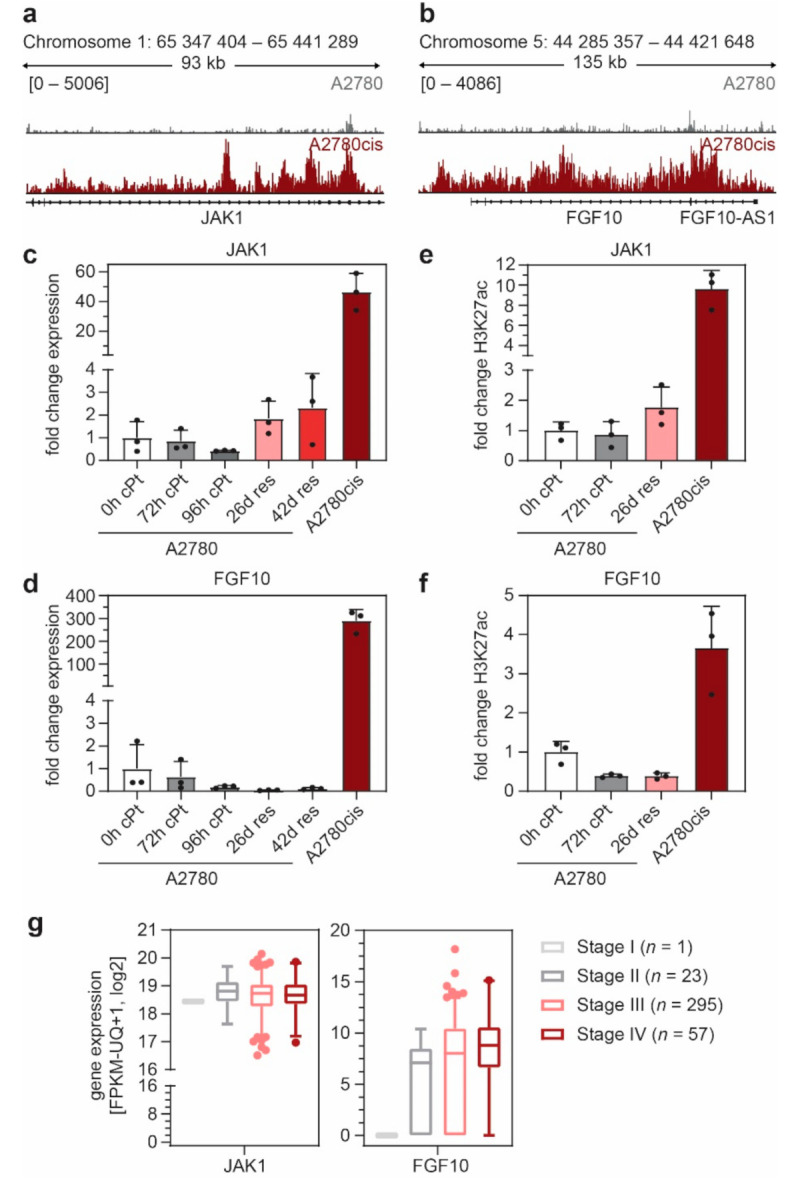
*JAK1* and *FGF10* acquire H3K27ac during cPt resistance development and are early and late markers for EOC development. (**a**,**b**) ChIP-seq tracks of H3K27ac intensity at the *JAK1* (A) and *FGF10* (B) gene in sensitive A2780 and resistant A2780cis cells. (**c**,**d**) Gene expression of *JAK1* (**c**) and *FGF10* (**b**) following cPt treatment (RT-qPCR) relative to *SDHA* control, normalized to untreated A2780 cells (0 h cPt). (**e**,**f**) H3K27ac signal at *JAK1* (**e**) and *FGF10* (**f**) following cPt treatment (ChIP-qPCR, relative to spike-in) normalized to untreated A2780 cells (0h cPt). (**c**–**f**) A2780 cells were treated with cPt (1 µM) for the indicated time points; 26d res and 42d res indicate cells which acquired resistance by treatment of A2780 cells with cPt (1 µM) for the indicated days. Mean ± SD, *n* = 3. (**g**) Analysis of *JAK1* and *FGF10* expression (RNA-seq) in patients with different FIGO stages of EOC from the GDC TCGA Ovarian Cancer (OV) cohort. Box plot with 2.5 and 97.5 percentile is shown for the indicated number of patient samples per group.

**Figure 4:**
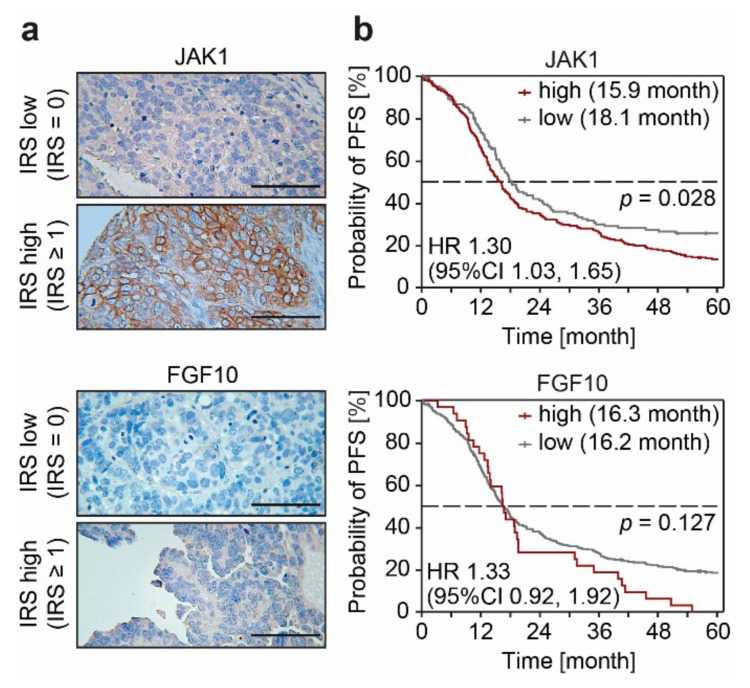
FGF10 and JAK1 can serve as independent predictive biomarkers. (**a**) Representative IHC images of EOC patient tissue stained for JAK1 or FGF10. IRS was used to attribute specimen according to their protein expression level for the Kaplan–Meier survival analysis shown in (**b**). Tissue samples with low (IRS = 0) or high (IRS ≥ 1) protein expression levels are shown (scale bar = 100 μm). (**b**) Kaplan–Meier survival analysis of progression-free survival (PFS) of 365 patients with high-grade serous EOC with low or high JAK1 or FGF10 expression level. Median survival of each group is indicated. Statistical significance was determined by Chi-Square statistics of the Log-Rank test (Mantel–Cox).

Development of resistance to cPt leads to the formation of a large enhancer cluster covering the entire region of the *JAK1* and *FGF10* gene in A2780cis cells (Figure 3a,b). This increase in H3K27ac is also reflected in the expression of both *JAK1* and *FGF10*, which differs substantially between cPt-sensitive and resistant cells (Figure 3c,d). We observed a gradual decrease in *JAK1* expression upon acute treatment of sensitive cells, which increased in short-term cPt-resistant A2780 cells (26d res and 42d res) (Figure 3c). In line with this, short-term treatment with cPt led to a decrease in H3K27ac signal at the *JAK1* promoter region, which then increased once A2780 cells acquired cPt resistance (Figure 3e). In contrast, expression of *FGF10* and H3K27ac signal intensity at the *FGF10* region were only elevated in the long-term resistant A2780cis cells (Figure 3d,f). To validate these findings, we extracted the gene-expression data for *JAK1* and *FGF10* from an independent RNA-seq dataset of other sensitive and resistant EOC cell lines (CCLE) [33] for which cPt GI_50_ information was available (Table S5). In line with our gene expression analysis, resistant cell lines showed a trend of higher *JAK1* and *FGF10* expression compared to sensitive cell lines (Figure S7a,b). To evaluate a possible predictive potential of *JAK1* and *FGF10* as biomarkers for patient stratification, we analyzed *JAK1* and *FGF10* gene expression in EOC patients within the publicly accessible TCGA database. Interestingly, expression of *JAK1* was high in all patients, whereas the expression of *FGF10* correlated positively with FIGO stage (Figure 3g). Although initial FIGO stage is not directly correlated with chemotherapy resistance in EOC in general, these data suggest that the expression of both genes might be involved in the response of patients to initial cPt treatment. Protein expression of JAK1 and FGF10 was further evaluated in patients with primary EOC by IHC (Figure 4a). Patients with high JAK1 expression had significantly decreased OS, as well as PFS. FGF10 expression was associated with significantly decreased OS, while PFS did not differ (Figure 4b and Figure S7c). OS data for EOC patients from the GDC TCGA Ovarian Cancer dataset support these findings (Figure S7d). In a multivariate analysis with known clinical prognostic factors for EOC, JAK1 was confirmed to be an independent prognostic marker for PFS and OS while FGF10 did not exhibit statistical independence (Table S7).

### 3.4. MBD3 and Components of the NuRD Complex Sensitize EOC Cells to Platinum Treatment 

**Figure 5:**
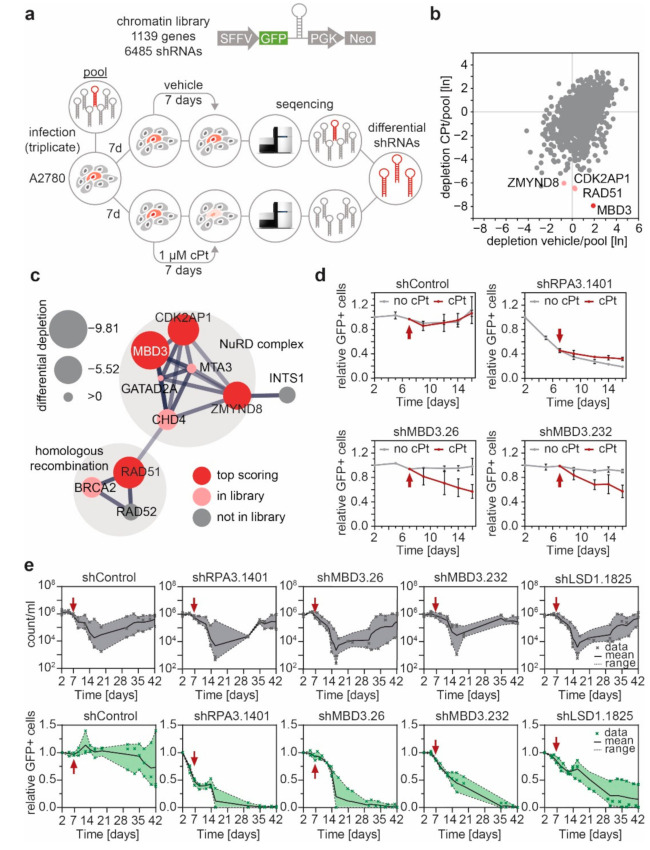
A multiplexed RNAi screen identifies MBD3 as a sensitizer to cPt treatment and suppressor of cPt resistance acquisition in A2780 EOC cells. (**a**) Schematic representation of the multiplexed RNAi screening strategy using the indicated chromatin-focused shRNA library. (**b**) Scatter plot showing the relative depletion (day 14/pool) of all genes in the library under cPt treatment compared to the vehicle control. The top-four depleted genes are highlighted. Geometric mean, *n* = 3. (**c**) String network analysis of the top four differential hits. Node radius indicates level of differential depletion. Red node color indicates a differential top hit, and light red indicates genes present in the library. Edges between nodes indicate both functional and physical protein associations, line thickness indicates the strength of the underlying data. (**d**) Competitive proliferation assays of A2780 EOC cells expressing the indicated shRNAs. Shown is the relative fraction of GFP^+^/shRNA^+^ cells relative to the initial measurement. After 7 days, cells were treated with vehicle or cPt (1 µM), as indicated by the red arrow, and analyzed for additional 9 days. Mean ± SEM, *n* = 3. (**e**) Competitive proliferation assays of A2780 EOC cells expressing the indicated shRNAs to determine the long-term effects of suppression of MBD3 or LSD1 expression under cPt treatment. A2780 cells expressing the indicated shRNAs were analyzed for the absolute cell number (count/mL) and the fraction of GFP^+^/shRNA^+^ cells relative to the initial measurement. Treatment with cPt (1 µM) was started, as indicated by the red arrow. Mean with range (shaded area) and individual data points are shown, *n* = 3.

Epigenetic aberrations are, in principle, reversible and dynamically regulated by a machinery that is amenable to pharmacologic intervention. Having found differential enhancers between sensitive and resistant cell lines that increased expression of clinically relevant genes, we aimed to identify regulatory molecules that could modulate the enhancer remodeling process and prevent cPt resistance development. To this end, we performed a multiplexed RNAi screen, using a microRNA-embedded short hairpin RNA (shRNAmir) library targeting chromatin regulators (Table S8) in combination with cPt treatment in the cPt-sensitive A2780 cell line (Figure 5a). Four genes were significantly depleted in the presence of cPt. Besides two core components of the NuRD complex, MBD3 and the Cyclin Dependent Kinase 2 Associated Protein 2 (CDK2AP2), we identified the recombinase RAD51 and Zinc Finger MYND–Type Containing 8 (ZMYND8) (Figure 5b and Figure S8a–c). A STRING network analysis revealed that these four proteins belong to two sub-complexes, namely the NuRD complex and another sub-complex consisting of components of the DNA repair machinery (Figure 5c), connected via an interaction between the Chromodomain-Helicase-DNA-Binding Protein 4 (CHD4) with RAD51 [46,47,48]. This is in line with recent literature connecting the NuRD complex to DNA damage response (DDR) in different cell types [16,49]. Suppression of the NuRD components GATA Zinc Finger Domain–Containing Protein 2A (GATAD2A), Metastasis-Associated Protein 3 (MTA3) and CHD4 and the DDR associated BRCA2 also showed depletion under cPt treatment in the multiplexed RNAi screen, but to a lesser extent (Figure 5c). Suppression of MBD3 expression with two independent shRNAs sensitized A2780 cells to cPt treatment (Figure 5d and Figure S8d,e). Long-term cultivation of A2780 cells in the presence of cPt showed, that after an initial drop in cell numbers, resistance was rapidly established after ~35 days. While cells expressing a neutral control shRNA (shControl) showed a similar proportion of surviving GFP+ cells at their resistant stage, cells expressing shRNAs targeting MBD3 or a validated shRNA [22] targeting the NuRD associated lysine-specific demethylase 1 (LSD1) disappeared over time, indicating that loss of NuRD components, MBD3 or LSD1, respectively, prevented the emergence of resistance in A2780 cells (Figure 5e).

### 3.5. Suppression of MBD3 Is Associated with Alterations of H3K27ac within Selected Regulatory Regions 

**Figure 6:**
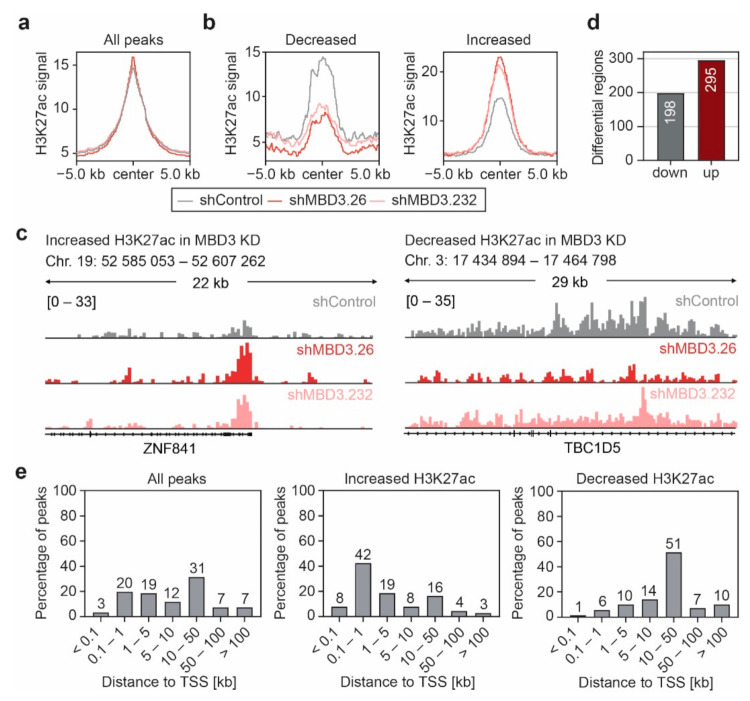
Suppression of MBD3 is associated with alterations of H3K27ac within selected regulatory regions. (**a**,**b**) Profile plot of the average H3K27ac ChIP-seq signal in A2780 cells expressing control or *MBD3* shRNAs for all H3K27ac peaks (**a**) or regions that show an increase or decrease of H3K27ac upon *MBD3* suppression (**b**). Regions with differential H3K27ac ChIP-seq signal were identified by differential peak filtering. Average H3K27ac signals are plotted with a 10 kb window around the peak center. (**c**) Representative ChIP-seq tracks of H3K27ac intensity for regions with increased or decreased H3K27ac signal in MBD3 KD A2780 cells from (**b**). (**d**) Number of genomic regions associated with increased or decreased H3K27ac signal upon *MBD3* suppression in A2780 cells. (**e**) Distribution of the H3K27ac peak distance to the nearest TSS identified by ChIP-Enrich.

MBD3 has been described as a bridging protein between two NuRD sub-complexes with distinct molecular functions thereby maintaining the structural integrity of NuRD [50]. The regulation of gene expression activity and DDR are two relevant functions of the NuRD complex which could lead to the observed anti-proliferative effect upon suppression of MBD3 in the presence of cPt. However, in immunofluorescence studies we did not observe any significant changes in DDR upon treatment with cPt in cells that express shRNAs targeting *MBD3* or *LSD1*, neither in the induction of DNA damage, documented by the deposition of γH2Ax (Figure S9a,c), nor in homologous recombination (HR), indicated by the formation of RAD51 foci (Figure S9b,d). These data suggest that suppression of *MBD3* expression does not lead to an impaired DDR in EOC. Several studies reported binding of the NuRD core complex components MBD3 or CHD4 to active promoters and enhancers decorated with H3K27ac [17,51,52]. Thus, we analyzed alterations in H3K27ac in A2780 cells upon suppression of MBD3 and observed only mild global effects with two independent shRNAs (Figure 6a). However, a select number of loci showed a distinct gain or loss of H3K27ac after suppression of *MBD3* (Figure 6b–d and Figure S10) in the close vicinity of the TSS or in 10–50 kb distance to the TSS, respectively (Figure 6e).

### 3.6. MBD3 Suppression Leads to Modulation of H3K27ac at Resistance Associated Enhancers 

**Figure 7:**
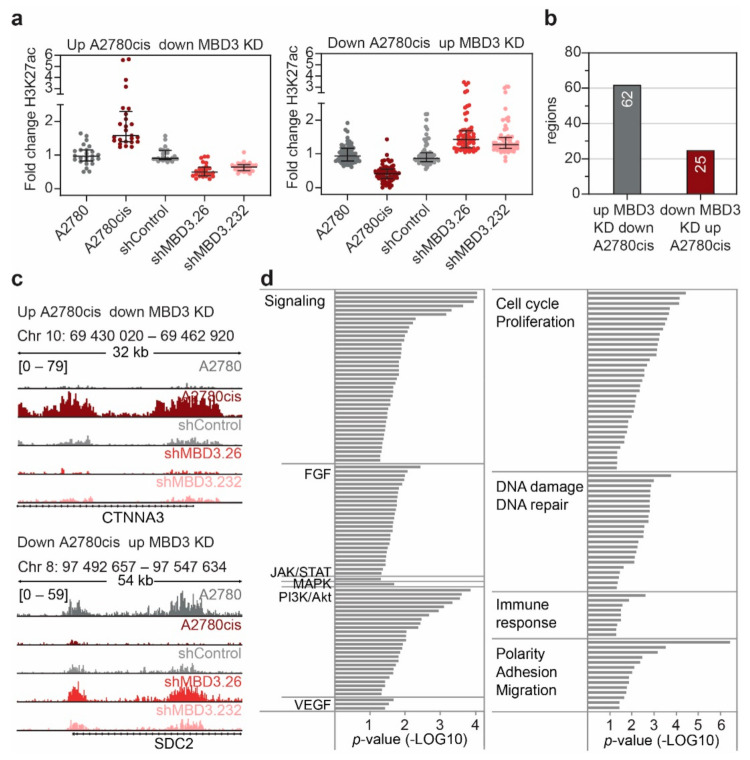
MBD3 suppression modulates H3K27ac in differential regions associated with resistance. (**a**) Scatter plot showing the fold change in H3K27ac signal of all regions with inverse change in H3K27ac signal between *MBD3* suppression and cPt-resistant A2780cis cells. Fold change is shown for regions with increased acetylation in resistant cells and decreased acetylation in sensitive A2780 cells (left) and regions which display decreased H3K27ac in resistant cells and increased H3K27ac in sensitive A2780 cells (right). Respective shRNAs are indicated. Median and interquartile range are shown. (**b**) Number of genomic regions associated with the differentially regulated regions identified in (**a**). (**c**) Representative ChIP-seq tracks of H3K27ac signal in A2780 and A2780cis cells and A2780 cells expressing the indicated shRNAs. (**d**) Gene-set-enrichment analysis (ChIP-Enrich) for all genes associated to the differentially regulated regions identified in (**a**). All identified pathways with a *p*-value ≤ 0.05 were categorized as indicated.

We aimed to evaluate whether MBD3 suppression leads to a modulation in H3K27ac at enhancers that are associated with the resistant phenotype in EOC. To this end, we identified H3K27ac regions showing the opposite response upon MBD3 suppression compared with cPt-resistant A2780cis cells (Figure 7a–c and Figure S11). Interestingly, suppression of MBD3 expression modulated H3K27ac at regions connected with FGF, VEGF, JAK/STAT, MAPK and PI3K/Akt signaling (Figure 7d and Table S9), which are associated with resistance and more aggressive disease in various cancers [39,53,54,55]. Furthermore, MBD3 suppression modulated the H3K27ac signal at genes associated with pathways that are known to be aberrantly activated in cancer, such as cell cycle and proliferation pathways, DNA damage and repair, immune response and EMT-associated pathways (Figure 7d and Table S9) [56,57,58,59]. Overall, this implies that interference with MBD3 expression and subsequently the stability of the NuRD complex results in a modulation of specific enhancers driving the expression of genes which are necessary for the development of cPt resistance.

## 4. Discussion

Platinum compounds are an essential part of first-line treatment in epithelial ovarian cancer (EOC) chemotherapy [2], but patients often develop resistance to the drug over time [4]. A better understanding of the processes underlying resistance development will help to improve the effectiveness of EOC therapy in the future and thus help to improve survival rates for patients with aggressive forms of EOC. To achieve this, we applied a multidimensional approach to investigate platinum (cPt) resistance mechanisms in EOC, combining epigenomic profiling of patient samples and cell lines with advanced functional genetic approaches. Analysis of the enhancer landscape (H3K27ac and H3K9ac) of two sensitive and resistant cell line pairs from different EOC subtypes (endometrioid ovarian adenocarcinoma and high-grade serous ovarian adenocarcinoma) showed distinct changes of both acetylation marks at regulatory enhancer elements (Figure 1; Supplementary Materials Figures S2 and S4). Differential H3K27ac regions were associated with changes in gene expression (Figure 2e). These genes were linked to Wnt, MAPK and FGF signaling, as well as pathways associated with proliferation, polarity, adhesion and migration (Figure 2d), which have been associated with increased resistance to treatment [39,56,58]. Our observations suggest that cPt treatment causes a genome-wide reorganization of the H3K27ac mark at distant regulatory elements. Most likely, transcriptional plasticity results in expression changes of crucial genes during the stress response, leading to resistance against platinum compounds [13,14]. In line with this hypothesis, we also observed that higher levels of H3K9ac and H3K27ac in patients correlated with reduced survival (Figure 1b). This suggests that higher levels of H3K9ac and H3K27ac promote the remodeling of enhancers due to enhanced transcriptional plasticity [60].

Recent genome-wide association studies (GWASs) combined with epigenomic profiling demonstrate that noncoding single nucleotide polymorphisms (SNPs) are frequently located in cell-type-specific enhancer elements [61,62]. Therefore, we investigated if prolonged treatment with cPt and subsequent DNA damage resulted in an increase in SNPs at these regulatory elements, which could explain the shutdown or re-establishment of enhancers at distinct genomic regions. We observed an increase in SNPs at differential clusters only in the cPt-resistant A2780cis cell line as compared to its cPt-sensitive partner A2780. The cPt-resistant CAOV4cis cells, which were established more recently and thus have been exposed to cPt for a shorter time, did not show this phenomenon (Figure S5). This suggests that the accumulation of SNPs at differential clusters in A2780cis occurs due to the prolonged cPt treatment. Interestingly, cPt is concentrated within nuclear condensates of the mediator complex, which contributes to drug pharmacodynamics and an increase in DNA damage at enhancer regions [63]. This implies that the accumulation of SNPs at regions associated with high histone acetylation in the cPt resistant A2780cis cells is the consequence of the enrichment of cPt within mediator condensates at enhancer regions, which form during the process of cPt resistance development. This suggests that the enhancer remodeling processes occurs at the epigenome level, probably driven by the concerted action of the DNA damage repair machinery and chromatin remodeling processes.

We identified *FGF10* and *JAK1* to be associated with a strong enhancer in the cPt-resistant cell line A2780cis, and both were among the genes showing the most profound changes in gene expression between sensitive and resistant cells (Figure 3). In this context, high expression of JAK1 and FGF10 was also associated with impaired overall survival in the investigated patient cohort (Figure 4 and Figure S7c,d) which for JAK1 was statistically independent and indicates its potential as a predictive biomarker in terms of patient survival and stratification. JAK/STAT signaling has been described as one of the major signaling pathways aberrantly activated in EOC and inhibition of JAK1 by small molecule inhibitors was shown to suppress cancer progression of EOC and other cancer types [44,64]. Activation of the JAK/STAT signaling pathway was shown to modulate cell intrinsic mechanisms, such as EMT, stemness and de-differentiation, through alterations of the cellular transcriptional state [65,66]. These adaptive reprogramming mechanisms increase the chance of a cell to adopt to different cues and are influenced by therapeutic pressure [67]. Each transcriptional cell state emerges from the combined action of thousands of active enhancers, and enhancer reprogramming is common during normal vertebrate development. Analogous, cancer cells are able to exploit similar mechanisms to remodel their enhancer landscape and thus initiate different enhancers in order to activate the expression of key genes essential for cell survival and proliferation, in our case genes associated with the JAK/STAT and FGF signaling pathways. Seizing control of developmental programs through enhancer remodeling is an emerging problem in cancer biology that enables non-genetic resistance to therapeutic treatment [21,60,68,69,70,71,72]. Enhancers are characterized by the presence of H3K27ac and histone acetyltransferases compete for H3K27 with the PRC2 complex. The PRC2 complex has an opposing function, leading to repression of enhancer activity by generating trimethylated H3K27 (H3K27me3) [73]. A recent publication showed that the activation of the JAK/STAT pathway leads to phosphorylation of enhancer of zeste 2 (EZH2) in a specific leukemia subtype. EZH2 is the catalytic subunit of PRC2, which promotes disassembly of the PRC2 complex leading to decreased global H3K27me3 levels [74]. This shifts the balance towards H3K27ac leading to activation of enhancers and promoters, conferring higher proliferative capacity of the affected cells. A comparable effect could be envisioned in OC, which needs to be experimentally proven in future studies.

Thus, JAK1 might be an interesting target for combinational treatment in EOC. In contrast, FGF10 has not previously been associated with EOC biology. We studied the changes in expression and H3K27ac deposition of *JAK1* and *FGF10* at different time points following the establishment of cPt resistance and noticed an increase in expression of *JAK1* in early resistant cells (Figure 3). This implies that *JAK1* may be involved in an immediate resistance response, whereas *FGF10* expression only increased in long-term resistant cells, indicating that FGF signaling might be established at a later stage in the resistance process (Figure 3).

We aimed to identify means to interfere with the process of enhancer remodeling and performed a combinatorial RNAi screen in the presence of cPt. This approach identified that *MBD3* and other components of the NuRD complex were involved in sensitizing A2780 cells to cPt. The NuRD complex plays a crucial role in many cellular processes through the regulation of transcription and has been implicated in oncogenesis and cancer progression, where it can facilitate or inhibit tumorigenesis [16]. MBD3 acts as a molecular bridge between different sub-complexes and maintains the structural integrity of the fully active NuRD complex [17,50]. This bridging function could explain why *MBD3* was the top hit identified in our screening approach [75]. Indeed, we observed that suppression of *MBD3* or *LSD1* (another NuRD subunit) sensitized A2780 cells to cPt and prevented the development of cPt-resistant A2780 cells even after long-term exposure (>35 days) with a GI_25_ dose of cPt (Figure 5e and Figure S2a). Although our data were generated in the EOC cell line A2780, which was established from an ovarian endometrioid adenocarcinoma tumor, and cannot be generalized to different EOC subtypes, several studies described that pharmacologic inhibition of NuRD components had a negative proliferative effect in OC cell lines of other subtypes [76,77,78,79,80]. Treatment with a LSD1 inhibitor was shown to sensitize EOC cells to cPt and upregulation of LSD1 in EOC patients is associated with poor prognosis [81]. Similarly, inhibition of histone deacetylases was shown to increase cPt sensitivity of high grade serous ovarian cancer cells [76]. Furthermore, inhibition of mediators of enhancer activity, such as BRD4, effectively sensitizes other EOC subtypes to cPt [82]. The NuRD complex has a well-described role in the response to DNA damage, and depletion of NuRD components causes a deficient DDR [83,84]. Therefore, we reasoned that the sensitization effect through suppression of *MBD3* was due to a non-functional NuRD complex, which failed to assist in the response to DNA damage. Surprisingly, we did not observe any alterations in γH2A.X or RAD51 loading upon suppression of *MBD3* or *LSD1* after treatment of A2780 cells with cPt (Figure S9). ZMYND8 and MBD3 have been shown to co-occupy active promoters and enhancers [51], and, indeed, we observed alterations in H3K27ac at distal regulatory regions upon suppression of *MBD3* expression (Figure 6), suggesting that MBD3 controls their activity. This, together with the fact that we did not observe alterations in DDR mechanisms upon suppression of *MBD3* expression, supports the hypothesis that the enhancer reprogramming we observe in EOC cell lines is a direct result of the DNA damage induced by treatment with cPt. For instance, p53 was shown to interact with NuRD complex components [85], and p53 binding events occur predominantly within regulatory enhancer elements mostly located within regions of inaccessible chromatin [86]. A large subset of these enhancers becomes accessible after DNA damage, indicating that p53 regulates these by modulating chromatin accessibility [86,87]. Most importantly, our second differential hit *RAD51* is associated with the NuRD complex [49,88] and has recently been described to be necessary for the maintenance of active enhancers in different cancer cell lines [89].

## 5. Conclusions

**Figure 8:**
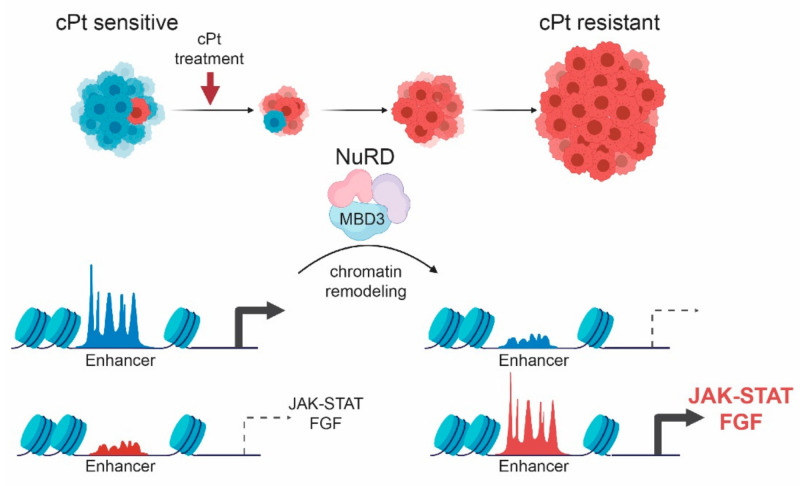
Model for the development of non-genetic resistance to platinum treatment in EOC. Upon prolonged treatment of EOC cells with cPt the initially sensitive cells develop into a cPt-resistant population. Resistance development leads to remodeling of the enhancer landscape, which is influenced by the NuRD complex and leads to differential expression of genes associated with key developmental signaling pathways, such as JAK/STAT and FGF signaling.

Previous studies have provided evidence that enhancer reprogramming is closely related to tumorigenesis and cancer development [90]. On the basis of the epigenomic profiling in cPt-sensitive and -resistant cell line pairs, we identified independent biomarkers for patient stratification to improve EOC prognosis. Moreover, our functional genetic-screening approach established candidates for new therapeutic regimes that sensitize tumors to chemotherapy and prevent the establishment of cPt resistance. We identified the NuRD complex to be responsible for the development of resistance in EOC and could show that acquisition of resistance to cPt is associated with enhancer remodeling. This leads to the expression of genes associated with the JAK/STAT and FGF signaling pathways, which were previously reported to be involved in resistance mechanisms to chemotherapeutic treatment (Figure 8). The data support the hypothesis that the establishment of cPt resistance is a result of enhancer remodeling induced by effective homologous recombination at sites of cPt-induced DNA damage. Increasing evidence supports this hypothesis, and previously published data demonstrate essential roles for transcriptional-mediated double-strand breaks in gene and enhancer activation [91,92,93,94]. Our study presents novel insights into the mechanism of acquired resistance to platinum treatment in EOC and potentially other cancers, thus ideally enabling the improvement of EOC patient treatment in the future.

## Data Availability

ChIP-seq data are available via Gene Expression Omnibus (GSE172510).

## Supplementary Materials

The following are available at https://www.mdpi.com/article/10.3390/cancers13153801/s1. Figure S1: H3K27 and H3K9 acetylation are associated with a decreased overall survival (OS) in EOC. Figure S2: Changes in H3K27ac in acquired resistance are correlated with changes in H3K9ac. Figure S3: Non-genetic resistance in EOC is associated with changes in the enhancer landscape. Figure S4: Non-genetic resistance in EOC is associated with changes in the enhancer landscape in CAOV4 cells. Figure S5: SNPs are accumulated in enhancer regions of long-term cPt-treated A2780cis cells. Figure S6: High gene expression does not correlate with super-enhancer status or H3K27ac level in general. Figure S7: FGF10 and JAK1 expression in EOC cell lines correlates with cPt GI50 values. Figure S8: MBD3 suppression sensitizes A2780 ovarian cancer cells to cPt treatment. Figure S9: Suppression of MBD3- or NURD-associated complex partners does not alter DNA damage response upon cPt treatment. Figure S10: MBD3 suppression induces H3K27ac alterations in defined regulatory regions. Figure S11: MBD3 suppression modulates H3K27ac in differential regions associated with resistance. Supplementary Material and Methods and Supplementary References. Supplementary Tables S1–S9.xlsx: Table S1: shRNAs used in this study. Table S2: Clinical characteristics of the 365 patients with first diagnosis of high-grade serous ovarian cancer evaluated by immunohistochemistry. Table S3: Primers used for RT-qPCR. Table S4: Primers used for ChIP-qPCR. Table S5: EOC cell lines of different subtypes and with available RNA-seq data and GI50 values for cPt. Table S6: Gene-set-enrichment analysis (ChIP-Enrich) for all genes associated with regions of increased (up) or decreased (down) H3K27ac in resistant A2780cis cells. Table S7: Multivariate analyses of prognostic factors. Table S8: Normalized reads chromatin-focused RNAi screen. Table S9: Gene-set-enrichment analysis (ChIP-Enrich) for all genes associated to regions with opposite change in H3K27ac signal between MBD3 suppression and A2780cis cells.
