# Supplementary material for: Functional Analysis of Non-Genetic Resistance to Platinum in Epithelial Ovarian Cancer Reveals a Role for the MBD3-NuRD Complex in Resistance Development
Source: Cancers (Basel). 2021 Jul 28;13(15):3801. doi: 10.3390/cancers13153801 (PMC8345099; doi:10.3390/cancers13153801)
Supplement: Supplementary file 1 [file cancers-13-03801-s001.zip › Supplementary Information_TB.pdf]

Functional analysis of non-genetic resistance to platinum in EOC reveals a role for the MBD3-NuRD complex in resistance development.

Tabea L. Bauer[1,2*], Katrin Collmar[1*], Till Kaltofen[2], Ann-Katrin Loeffler[1], Lorena Decker[1], Jan Mueller[1+], Sabine Pinter[1], Stephan A. Eisler[3], Sven Mahner[2], Patricia Fraungruber[2], Stefan Kommoss[4], Anette Staebler[5], Lewis Francis[6], R. Steven Conlan[6], Johannes Zuber[7,8], Udo Jeschke[2,9], Fabian Trillsch[2#], Philipp Rathert[1#]

[1] Department of Biochemistry, Institute of Biochemistry and Technical Biochemistry, University of Stuttgart, 70569 Stuttgart, Germany.

[2] Department of Obstetrics and Gynecology, University Hospital, LMU Munich, 81377 Munich, Germany

[3] Stuttgart Research Center Systems Biology (SRCSB), University of Stuttgart, 70569 Stuttgart, Germany.

[4] Department of Women's Health, Tubingen University Hospital, 72076 Tuebingen, Germany

[5] Institute of Pathology and Neuropathology, Tuebingen University Hospital, 72076 Tuebingen, Germany

[6] Swansea University Medical School, Singleton Park, Swansea, SA2 8PP, Wales, UK

[7] Research Institute of Molecular Pathology, Vienna BioCenter, Vienna, Austria.

[8] Medical University of Vienna, Vienna BioCenter (VBC), Vienna, Austria.

[9] Department of Obstetrics and Gynecology, University Hospital Augsburg, 86156 Augsburg, Germany

[+] present address: Department of In-vitro Diagnostics, Fraunhofer Institute for Interfacial Engineering and Biotechnology IGB, Stuttgart, Germany.

* These authors contributed equally to this work.

# To whom correspondence should be addressed:

Philipp Rathert (philipp.rathert@ibtb.uni-stuttgart.de) & Fabian Trillsch (fabian.trillsch@med.uni-muenchen.de)

Supplementary Material

Supplementary Figure S1: H3K27 and H3K9 acetylation are associated with a decreased OS in EOC.

Supplementary Figure S2: Changes in H3K27ac in acquired resistance are correlated with changes in H3K9ac.

Supplementary Figure S3: Non-genetic resistance in EOC is associated with changes in the enhancer landscape.

Supplementary Figure S4: Non-genetic resistance in EOC is associated with changes in the enhancer landscape in CAOV4 cells.

Supplementary Figure S5: SNPs are accumulated in enhancer regions of long term cPt treated A2780cis cells.

Supplementary Figure S6: High gene expression does not correlate with super-enhancer status or H3K27ac level in general.

Supplementary Figure S7: FGF10 and JAK1 expression in EOC cell lines correlates with cPt GI_{50} values.

Supplementary Figure S8: MBD3 suppression sensitizes A2780 ovarian cancer cells to cPt treatment.

Supplementary Figure S9: Suppression of MBD3 or NURD associated complex partners does not alter DNA damage response upon cPt treatment.

Supplementary Figure S10: MBD3 suppression induces H3K27ac alterations in defined regulatory regions.

Supplementary Figure S11: MBD3 suppression modulates H3K27ac in differential regions associated with resistance.

Supplementary Material and Methods

Supplementary References

SupplementaryTablesS1-S9.xlsx:

Supplementary Table S1: shRNAs used in this study

Supplementary Table S2: Clinical characteristics of the 365 patients with first diagnosis of high-grade serous ovarian cancer evaluated by immunohistochemistry.

Supplementary Table S3: Primers used for RT-qPCR

Supplementary Table S4: Primers used for ChIP-qPCR

Supplementary Table S5: EOC cell lines of different subtypes and with available RNA-seq data and GI_{50} values for cPt.

Supplementary Table S6: Gene set enrichment analysis (ChIP-Enrich) for all genes associated with regions of increased (up) or decreased (down) H3K27ac in resistant A2780cis cells.

Supplementary Table S7: Multivariate analyses of prognostic factors.

Supplementary Table S8: Normalized reads chromatin focused RNAi screen

Supplementary Table S9: Gene set enrichment analysis (ChIP-Enrich) for all genes associated to regions with opposite change in H3K27ac signal between MBD3 suppression and A2780cis cells.

Supplementary Figures

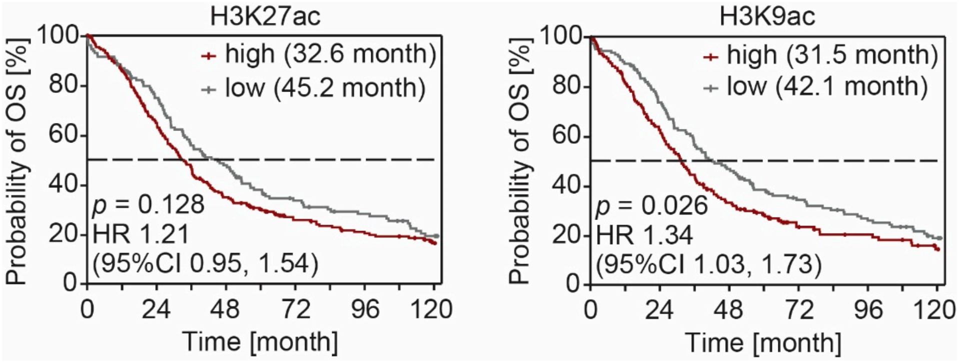

Supplementary Figure S1: H3K27 and H3K9 acetylation are associated with a decreased -overall survival (OS) in EOC. Kaplan-Meier survival analysis for progression-free survival (PFS) of 365 patients with high-grade serous EOC with low (IRS < 6) or high (IRS ≥ 6) H3K27ac or H3K9ac modification levels. Median OS of each group is indicated. Statistical significance was determined by Chi-Square statistics of the Log-Rank test (Mantel-Cox).

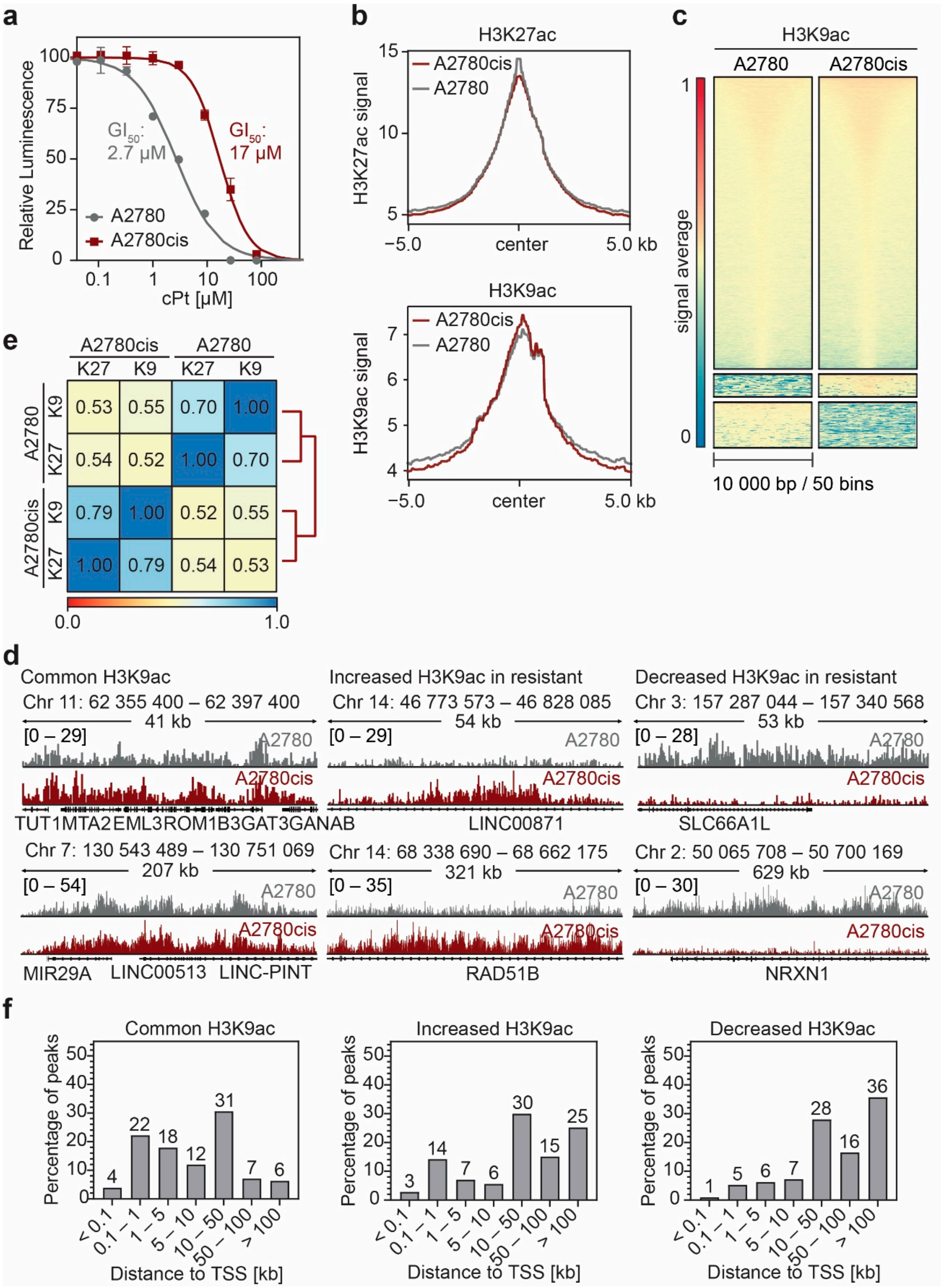

Supplementary Figure S2: Changes in H3K27ac in acquired resistance are correlated with changes in H3K9ac. (**a**) Dose-response curve of A2780 and A2780cis cells to treatment with different concentrations of cPt for 72h to determine cPt sensitivity. Cell viability was determined

relative to untreated control cells using CellTiter-Glo. Mea ±SD, n = 3 independent repeats, lines show a non-linear fit of the data. (**b**) Profile plot of average H3K27ac or H3K9ac ChIP-seq signal for all H3K9ac or H3K27ac peaks in A2780 and A2780cis cells. Average signals are plotted with a 10 kb window around the peak center. (**c**) Heatmaps showing the average H3K9ac ChIP-seq signal for A2780 and A2780cis EOC cells. Regions shown are the common and differential regions identified in the H3K27ac ChIP-seq data in Figure 1C. Signals are sorted by the highest average signal in A2780 cells and plotted on the heatmap with a 10 kb window around the peak center. (**d**) Representative ChIP-seq tracks of H3K9ac intensity in A2780 and A2780cis cells for regions with common and differential H3K9ac peaks. (**e**) Spearman correlation of H3K9ac and H3K27ac ChIP-seq signal in A2780 and A2780cis cells for all regions with H3K27ac signal. Spearman correlation coefficients are indicated. (**f**) Distribution of the distance of the common and differential H3K27ac peaks to the nearest TSS identified by ChIP-Enrich.

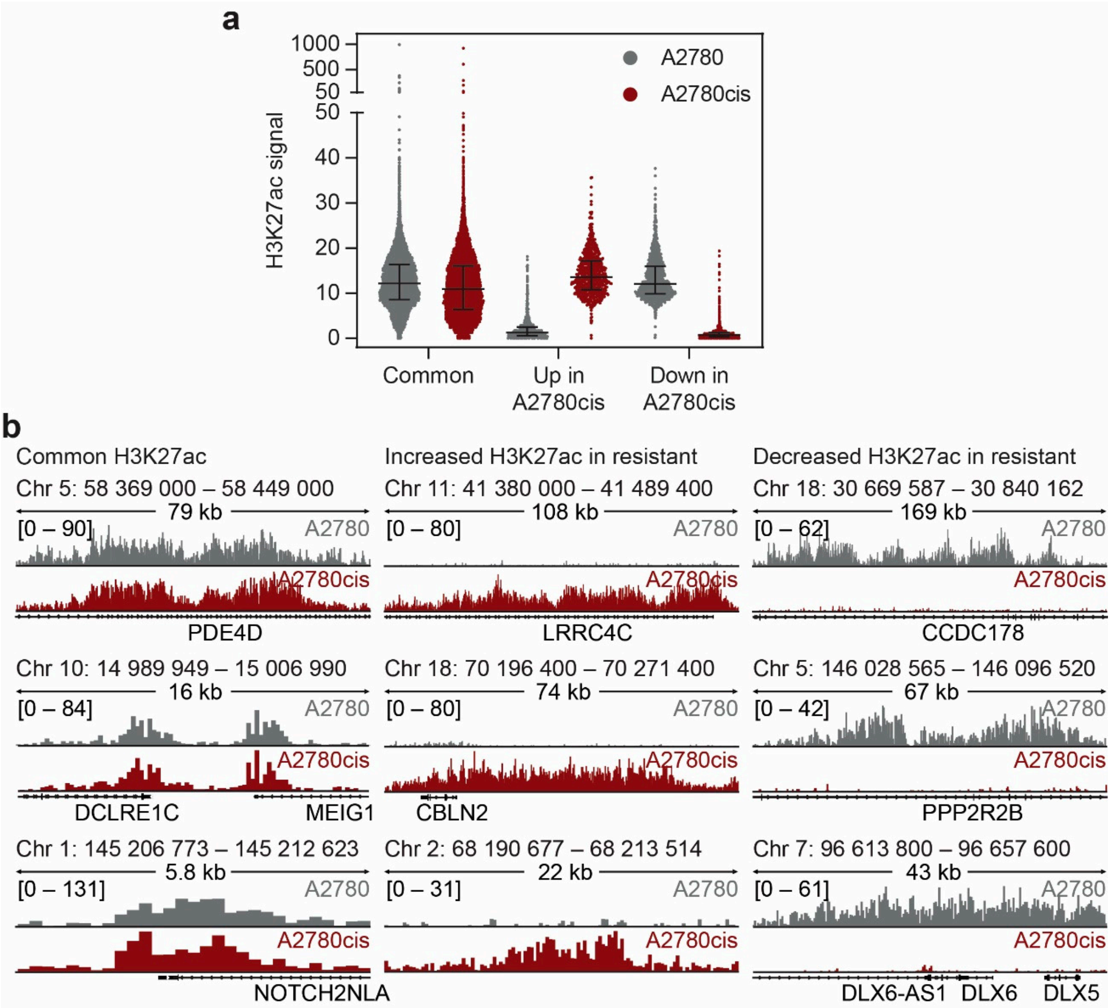

Supplementary Figure S3: Non-genetic resistance in EOC is associated with changes in the enhancer landscape. (**a**) Scatter dot plot of average H3K27ac signal of all common and differential regions identified in Fig 1C in A2780 and A2780cis cells. Average H3K27ac signal for all regions in A2780 and A2780cis cells was retrieved from bigwig files. Median and interquartile range are shown. (**b**) Representative ChIP-seq tracks of H3K27ac intensity in A2780 and A2780cis cells for regions with common and differential H3K27ac identified in Figure 1c.

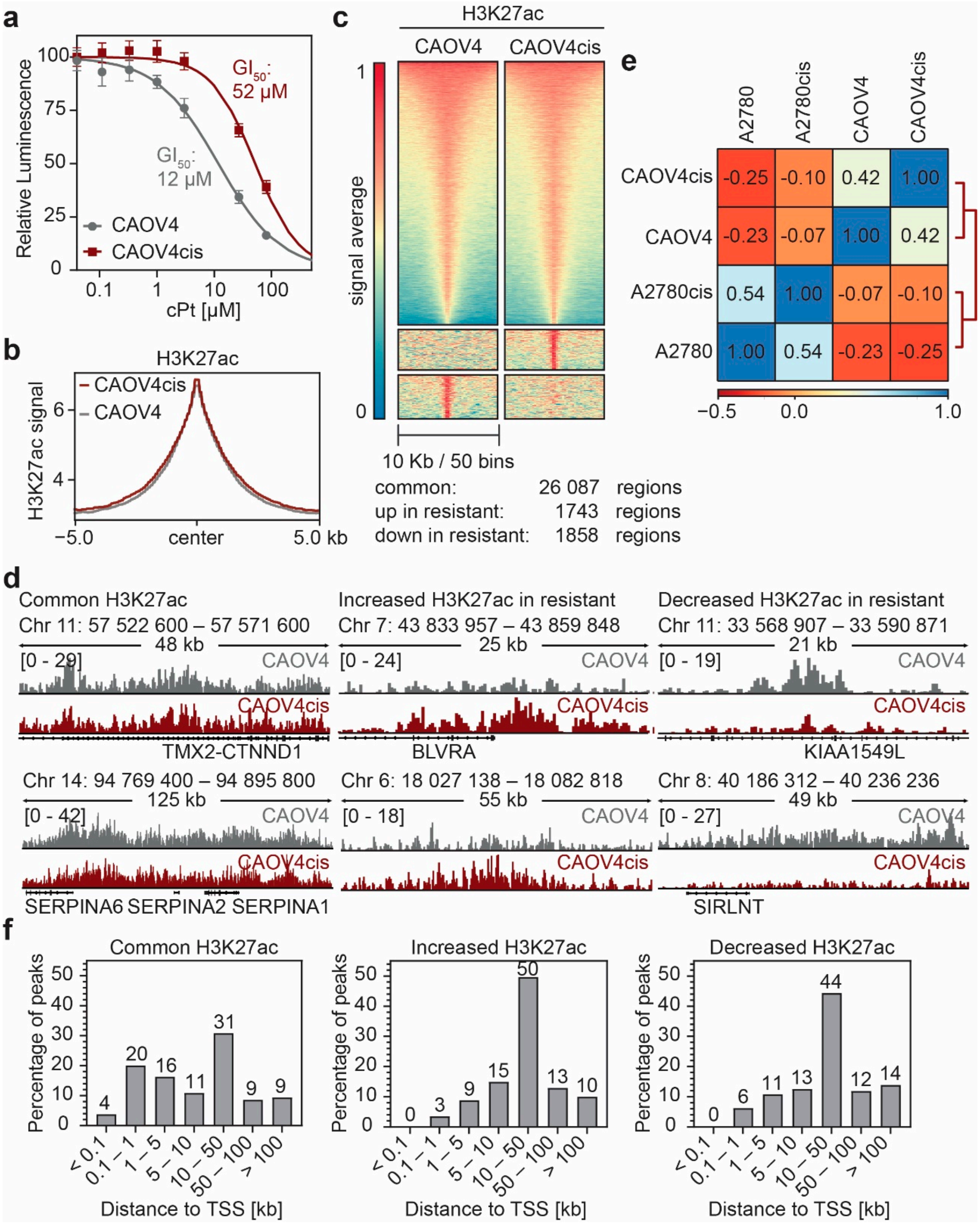

Supplementary Figure S4: Non-genetic resistance in EOC is associated with changes in the enhancer landscape in CAOV4 cells. (**a**) Dose-response curve of CAOV4 and CAOV4cis cells to treatment with different concentrations of cPt for 72h to determine cPt sensitivity. Cell viability was determined relative to untreated control cells by CellTiter-Glo. Mean±SD, n=3, lines show non-linear fit of the data. (**b**) Profile plot of the average H3K27ac ChIP-seq signal for all identified H3K27ac peaks in CAOV4 and CAOV4cis cells. Average signals are plotted with a 10 kb window around the peak center. (**c**) Heatmaps showing the average H3K27ac ChIP-seq signal for CAOV4

and CAOV4cis EOC cells. Signals are sorted by the highest average signal in CAOV4 cells and plotted on the heatmap with a 10 kb window around the peak center. Differential regions between the sensitive and resistant cells were identified by K-means clustering. (**d**) Representative ChIP-seq tracks of H3K27ac intensity in CAOV4 and CAOV4cis cells for regions with common and differential H3K27ac peaks identified in (c). (**e**) Spearman correlation of H3K27ac signal in A2780, A2780cis, CAOV4 and CAOV4cis cells. For correlation analysis regions identified for all four cells lines were combined. Spearman correlation coefficients are indicated. (**f**) Distribution of the distance of the common and differential H3K27ac peaks identified in (c) to the nearest TSS as identified by ChIP-Enrich.

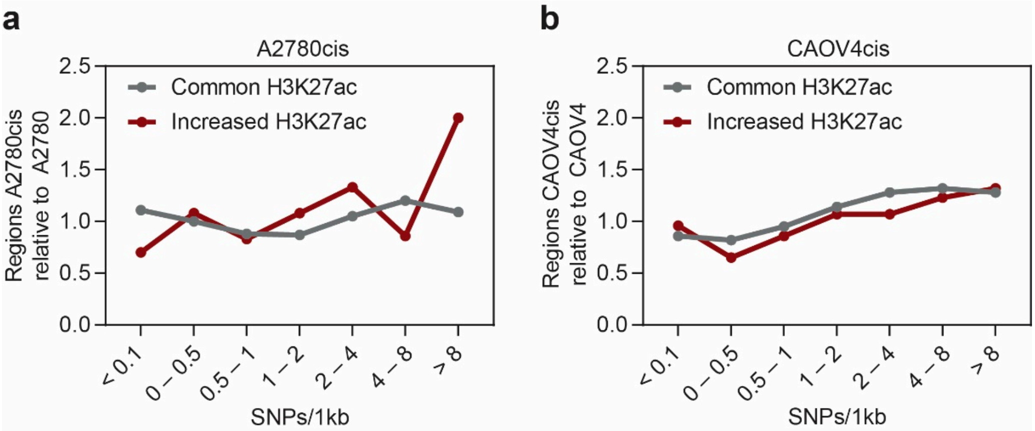

Supplementary Figure S5: SNPs are accumulated in enhancer regions of long term cPt treated A2780cis cells. (**a**) and (**b**) Frequency of SNPs per 1 kb in resistant A2780cis (a) and CAOV4cis (b) cells relative to their sensitive counterpart A2780 (a) or CAOV4 (b). SNPs in the common and differential regions were extracted from H3K27ac ChIP-seq data sets. Due to the low number of reads, regions with decreased H3K27ac were not considered. SNPs were identified using a Bayesian genetic variant detector and the number of SNPs relative to the length of the region (SNP frequency, SNPs/1 kb) for each region determined. To assess difference in the distribution of SNP frequencies between the sensitive and resistant cells, the occurrence of each SNP frequency in the resistant cells is shown relative to the sensitive cells.

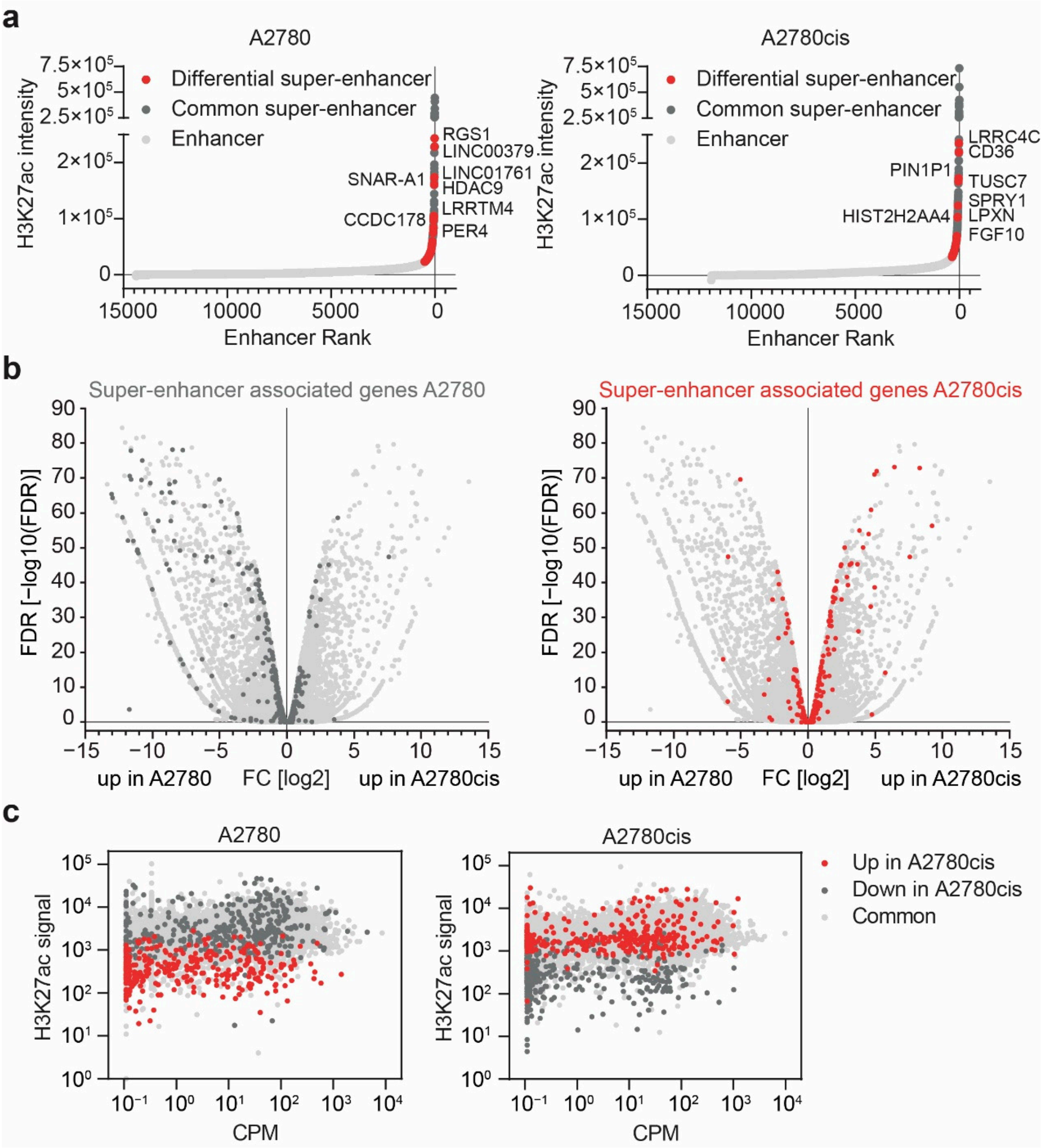

Supplementary Figure S6: High gene expression does not correlate with super-enhancer status or H3K27ac level in general. (**a**) Identification of super-enhancer in A2780 and A2780cis cells. Dot plot of enhancers ranked by H3K27ac signal using the ROSE algorithm. Super-enhancers identified by ROSE were filtered for super-enhancers that are common and differential between the A2780 and A2780cis cells. (**b**) Volcano plot displaying fold change of gene expression between sensitive A2780 and resistant A2780cis cells. Fold change of mean gene expression for all genes with H3K27ac ChIP-seq signal in A2780 or A2780cis cells was determined and plotted against their respective FDR value. Genes associated with super-enhancers in A2780 and A2780cis cells are highlighted. (**c**) Dot plot of gene expression versus cumulative H3K27ac intensity in sensitive A2780 and resistant A2780cis cells. Read count was converted into counts per million (CPM) by

edgeR and mean CPM for each gene determined. Genes associated with common and differential regions (H3K27ac signal up or down in resistant cells) are highlighted.

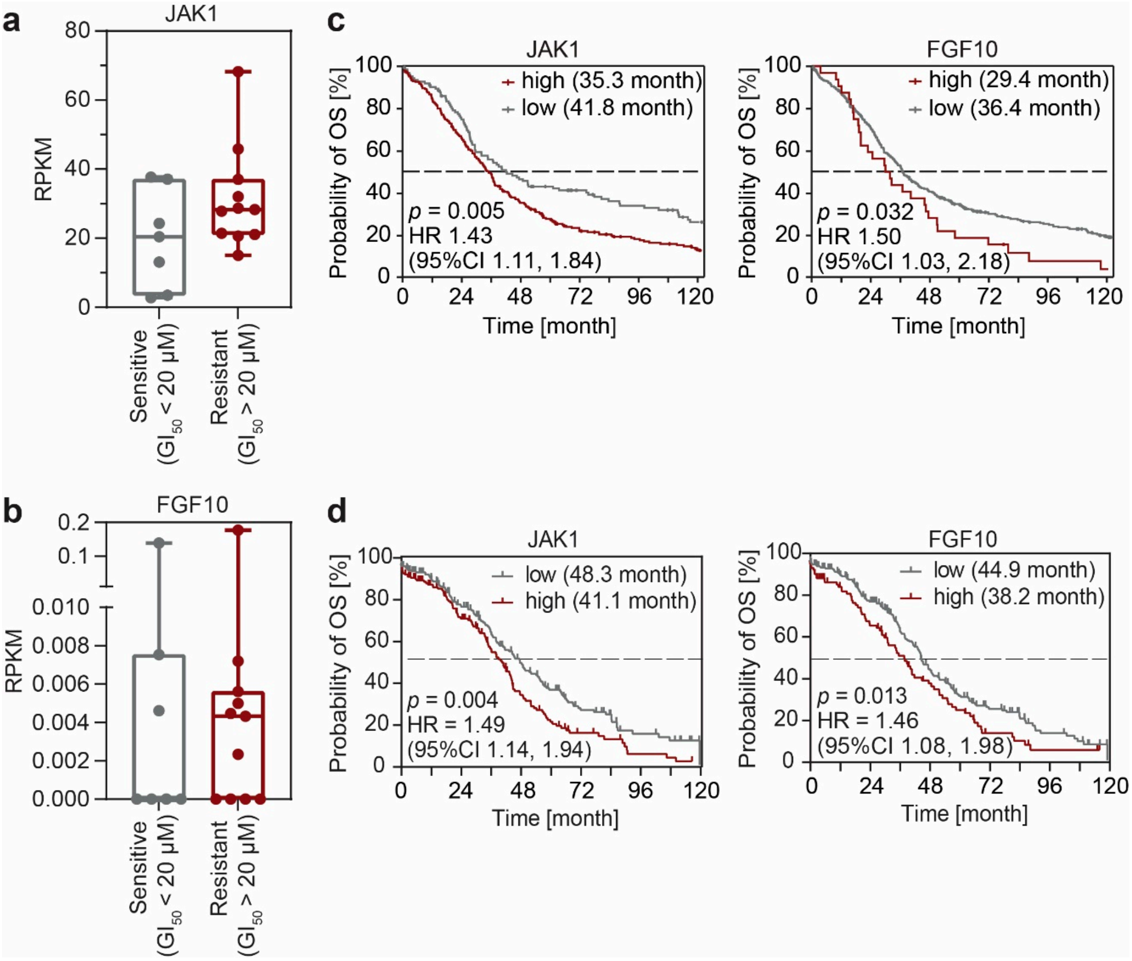

Supplementary Figure S7: FGF10 and JAK1 expression in EOC cell lines correlates with cPt GI_{50} values. (**a**) and (**b**) Gene expression of *JAK1* and *FGF10* in cPt sensitive and resistant EOC cells lines. Gene expression data for *JAK1* and *FGF10* was curated from an independent RNA-seq data set of EOC cell lines (CCLE) with available cPt GI_{50} values in the GDSC2 dataset. Expression data was plotted for all cell lines showing a $GI_{50} > 20$ µM (resistant, 11 total) or a $GI_{50} < 20$ µM (sensitive, 7 total). Data is shown as box plot with all data points plotted. (**c**) Kaplan-Meier survival analysis for overall survival (OS) of 365 patients with high-grade serous EOC with low or high JAK1 or FGF10 expression level. Median survival of each group is indicated. Statistical significance was determined by Chi-Square statistics of the Log-Rank test (Mantel-Cox). (**d**) Kaplan-Meier survival analysis for OSof 370 EOC patients with low (FPKM < 18.16) or high (FPKM ≥ 18.16) *JAK1* expression and low (FPKM < 0.06) or high (FPKM ≥ 0.06) *FGF10* expression. Data was taken from the GDC TCGA Ovarian Cancer (OV) cohort. Median survival of each group is indicated. Statistical significance was determined by Log-rank (Mantel-Cox) testing, hazard ratios following the Mantel-Haenszel's method.

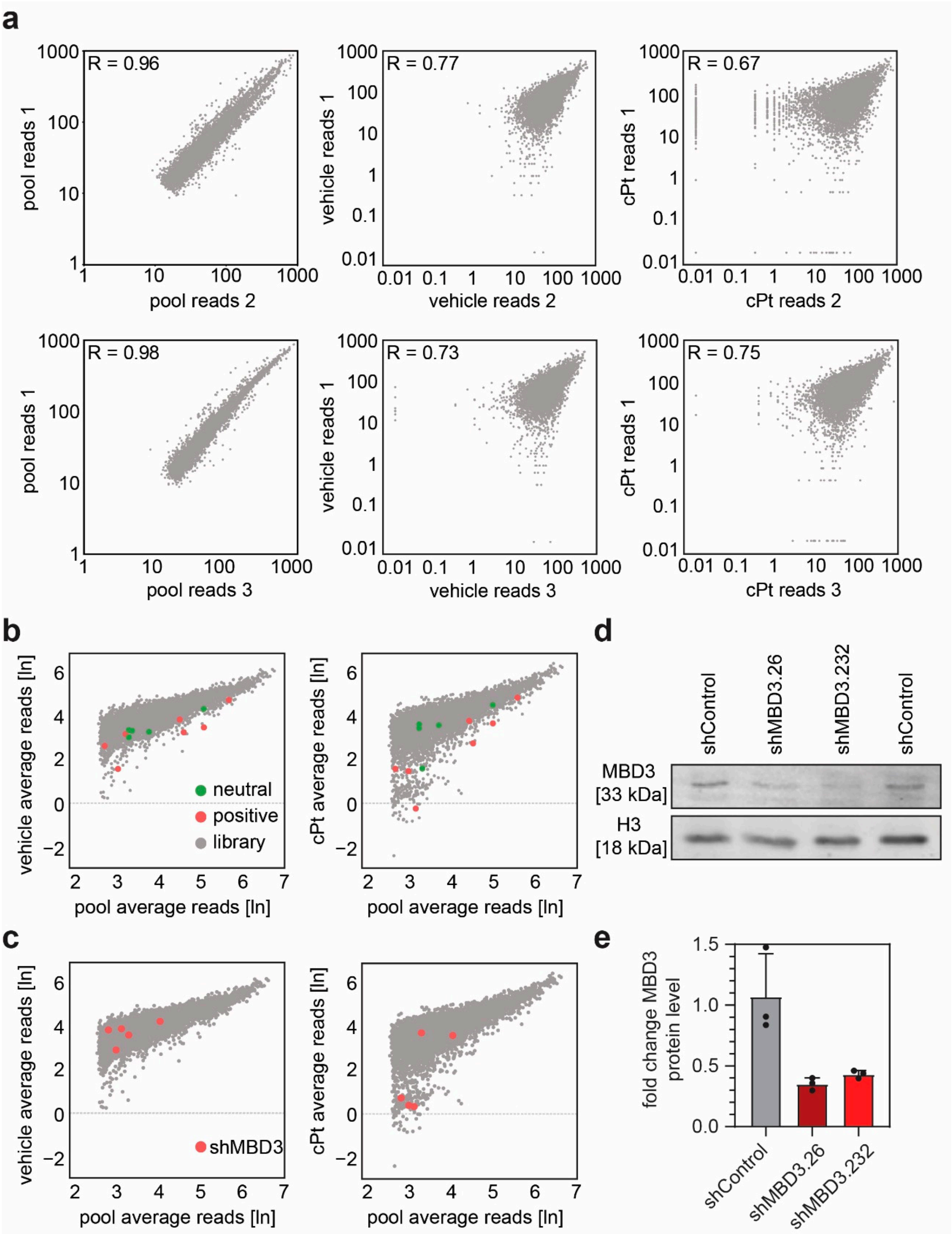

Supplementary Figure S8: MBD3 suppression sensitizes A2780 ovarian cancer cells to cPt treatment. (**a**) Reproducibility of the multiplexed RNAi screen using a chromatin-focused shRNA library. Raw reads for each condition and repetition are plotted together for comparison. Pearson's correlation coefficient is shown. (**b**) and (**c**) Multiplexed RNAi screen using a chromatin-focused shRNA library. Logarithm of average reads of pool is plotted over average

reads of vehicle or cPt treated cells. Reads of neutral control shRNAs, positive control shRNAs or shRNAs belonging to the library (b) and shRNAs targeting *MBD3* (c) are highlighted. (**d**) Validation of *MBD3* suppression by western blot. A2780 cells expressing the indicated shRNAs for 7 days were sorted for GFP positive (shRNA expressing) cells and analyzed for MBD3 protein levels by SDS-PAGE and western blotting. ß-Actin was used as loading control. One representative experiment out of 3 independent experiments for each shRNA is shown. (**e**) Fold change in MBD3 protein level upon *MBD3* suppression relative to shControl determined by western blot densitometry. Intensities were normalized to the loading control. Mean±SD of n=3.

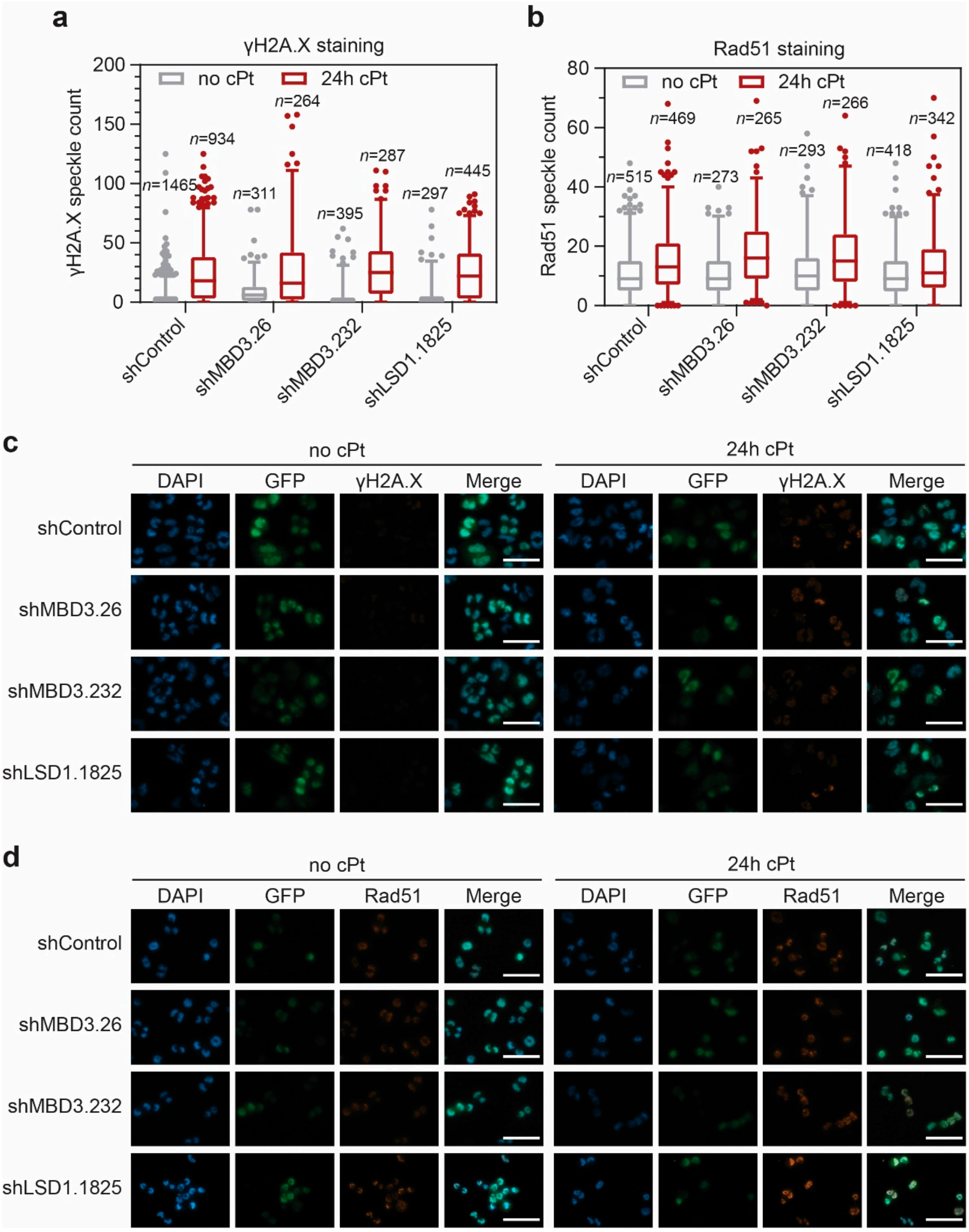

Supplementary Figure S9: Suppression of MBD3 or NURD associated complex partners does not alter DNA damage response upon cPt treatment. (**a**) and (**b**) Immunostaining of γH2A.X (a) or Rad51 (b) to determine DNA damage response upon cPt treatment and *MBD3* or *LSD1* suppression. A2780 cells expressing the indicated shRNAs for 7 days were split into two populations and treated with vehicle or cPt (1 μM). After 24 h of treatment, immunostaining with antibodies for the DNA damage repair markers γH2A.X or RAD51 was performed. Number of

speckles per nucleus for indicated number of nuclei were determined from $n \geq 2$ independent experiments and are shown as box plot with 2.5 and 97.5 percentile. (**c**) and (**d**) Representative single channel immunofluorescence images of the γH2A.X (c) or Rad51 (d) immunostaining (scale bar 50 μm).

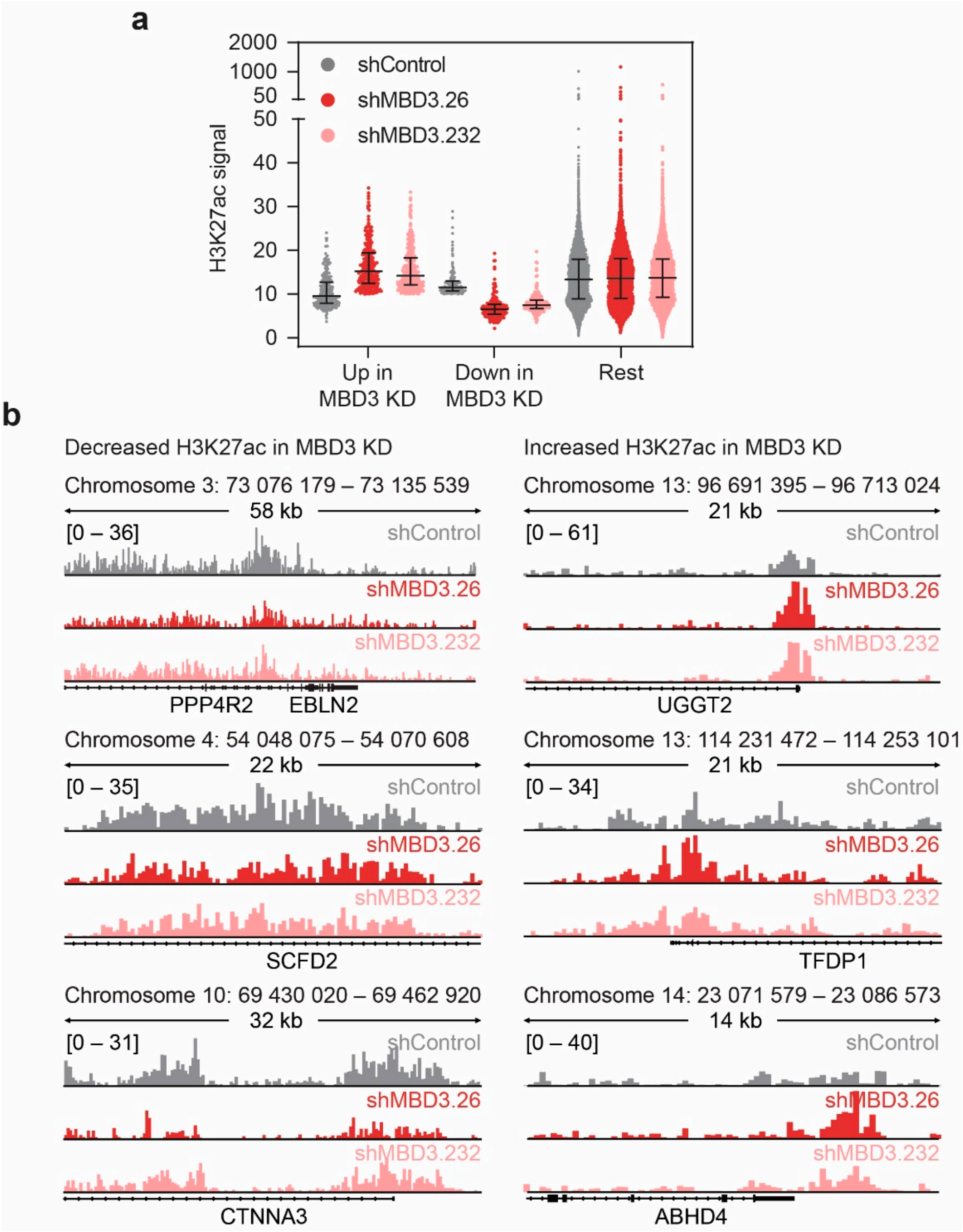

Supplementary Figure S10: MBD3 suppression induces H3K27ac alterations in defined regulatory regions. (**a**) Scatter dot plot of average H3K27ac signal of regions with increased, decreased or similar acetylation upon *MBD3* suppression in sensitive A2780 cells. Average H3K27ac scores for each region were devised from bigwig files. Median and interquartile range are shown. (**b**) Representative ChIP-seq tracks for regions with differential H3K27ac signal between shControl and *MBD3* suppression A2780 cells.

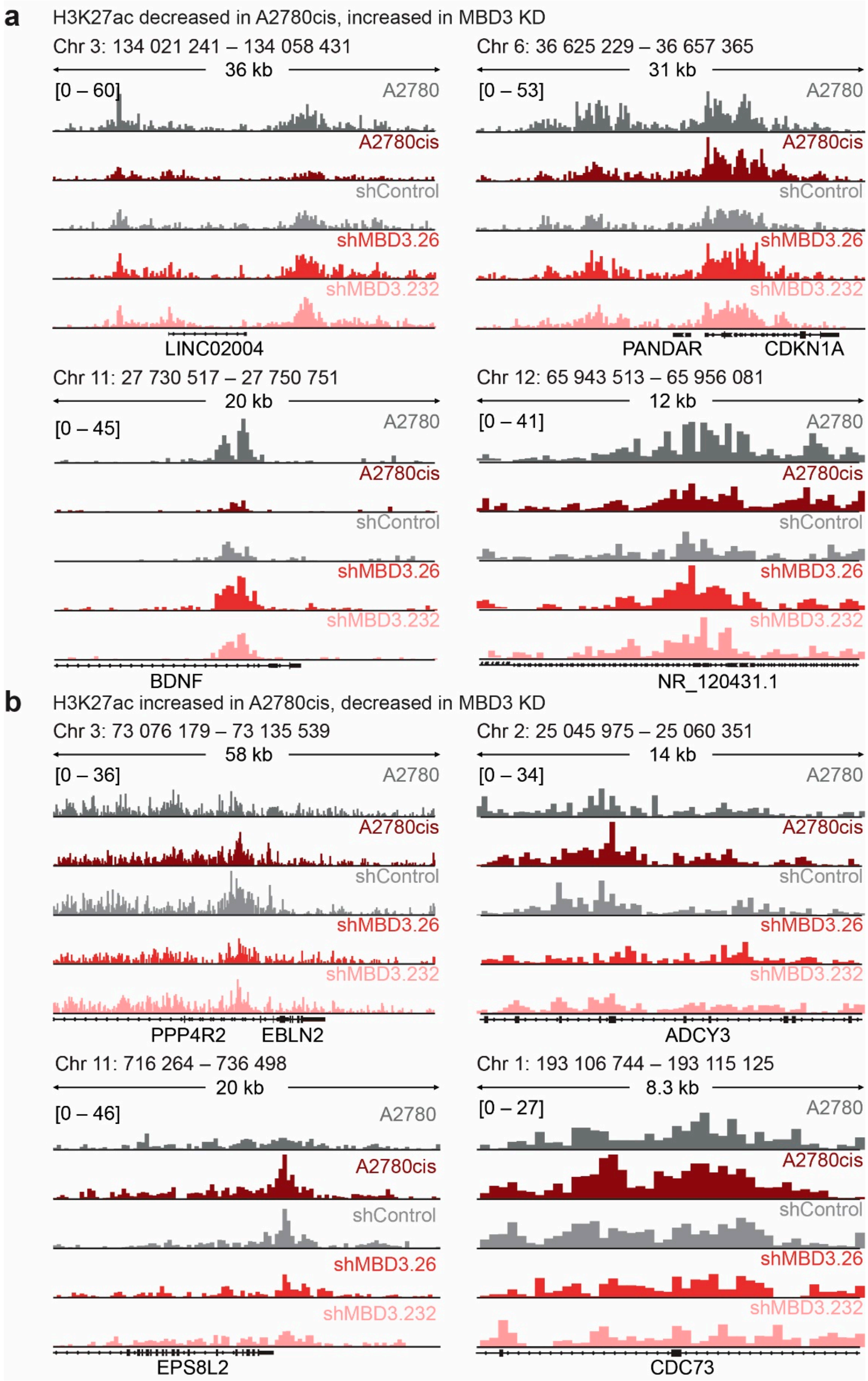

Supplementary Figure S11: MBD3 suppression modulates H3K27ac in differential regions associated with resistance. (**a**) and (**b**) Representative ChIP-seq tracks for regions that display

decreased H3K27ac in resistant cells but are increased in H3K27ac upon *MBD3* suppression in sensitive cells (a) and regions that show increased H3K27ac in resistant cells but decreased H3K27ac in sensitive A2780 upon suppression of *MBD3* expression (b).

Supplementary Material and Methods

Patient samples for Immunohistochemistry

For immunohistochemistry, formalin-fixed and paraffin-embedded (FFPE) tissue specimens of patients with first diagnosis of EOC undergoing primary radical cytoreductive surgery was analyzed. No inclusion or exclusion criteria were applied.

To generate hypotheses for further analyses, a cohort of 156 patients with EOC who underwent radical cytoreductive surgery at the Department of Obstetrics and Gynecology of the LMU Clinic between 1990 and 2002 was stained and evaluated for the endpoint OS (data not shown). Histopathological diagnoses were established by specialized gynecologic pathologist with staging and grading according to TNM and FIGO (International Federation of Gynecology and Obstetrics) classification. 90.0% of patients with high-grade serous EOC presented with advanced stage FIGO III-IV, while 10.0% were diagnosed in early stage disease (FIGO I-II). All patients received a platinum-based chemotherapy. Follow-up data was retrieved from the Munich Cancer Registry and aftercare calendars. Median age at primary diagnosis was 63.2 years. This study has been approved by the ethics committee of Ludwig Maximilian University of Munich (reference number 138/03 and 17/471).

In a second step and to confirm the obtained hypotheses in a larger and more recent cohort, a well characterized and homogenously treated cohort of 365 patients with high-grad serous ovarian cancer who underwent radical cytoreductive surgery between 2000 and 2014 in the Department of Women's Health of the Tuebingen University Hospital was evaluated for protein expression and histone modifications. Histopathological diagnoses were established by specialized gynecologic pathologist with staging and grading according to TNM and FIGO classification. 89.9% of patients presented with FIGO III-IV, while only 9.9% were diagnosed in early disease (FIGO I-II). 72.6% of the patients received a platinum-based chemotherapy. Lifetime data were taken from the Tuebingen Cancer Registry and aftercare calendars. Median age at primary diagnosis was 64.0 years. A summary of further patient characteristics can be found in Supplementary Table S1. This study has been approved by the ethics committee of University of Tuebingen. All histopathological analyses were carried out in compliance with the guidelines of the Helsinki Declaration of 1964 (last revision October 2018). No randomization was applied.

Sampling and Microarray Construction

Following pathologic evaluation, three core biopsies with 0.6 mm in diameter for each EOC patient were taken from representative regions of the FFPE tumor blocks. The biopsies were assembled in tissue microarrays (TMA) paraffin blocks by using a micro tissue arrayer (Beecher Instruments). Those TMA paraffin blocks were cut into serial sections of 5 µm which were then transferred and fixed on microscope slides. A haematoxylin and eosin staining were performed and evaluated to verify that representative areas of the tumor were aligned on the slides.

Immunohistochemistry (IHC) for tissue microarrays

IHC was carried out using a combination of pressure cooker heating and the ZytoChem-Plus HRP Polymer-Kit (Zytomed Systems) with 3,3'-diaminobenzidine (Roth) as chromogenic substrate according as previously described (1). Staining were performed with anti-Histone H3 (acetyl K27) antibody (1:2000), anti-Histone H3 (acetyl K9) antibody (1:200), anti-FGF10 antibody (1:100) or anti-JAK1 antibody (1:100). Evaluation, imaging and storing was done with an AxioScope microscope (Zeiss), an AxioCam digital camera system (Zeiss) and the AxioVision software (Zeiss). Immunohistochemical staining was assessed semi-quantitatively in a blinded fashion by two investigators, according to Remmele and Steger (1) using the IHC score (IR score, IRS or Remmele score; mean±SEM). It is calculated by multiplying a score representing the percentage of positively stained cells (0: no staining, 1: <10%, 2: 10-50%, 3: 51-80%, 4: >80%) with a score representing the cells staining intensity (0: none, 1: weak, 2: moderate, 3: strong) resulting in an IRS between 0-12. For the cytoplasmic staining of FGF10 and JAK1, absent expression with an IRS of 0 was opposed to expression with IRS ≥1. For nuclear staining of H3K27ac and H3K9ac, an IRS between 0-5 was regarded as low, while an IRS≥6 was considered as high modification levels.

Generation of Tet-on competent cells and lentiviral transduction

Generation of Tet-on competent cells. All cell lines used were modified to express the ecotropic receptor (EcoR) and the rtTA3 transcriptional activator for the Tet-on system using retroviral transduction. 70-80% confluent LentiX cells in supplemented DMEM were transfected with the pRRL.RiEP plasmid (pRRL.SFFV-rtTA3-IRES-EcoR-PGK-Puro, gift from Johannes Zuber) and the two packaging vectors pCMVR8.74 (gift from Didier Trono, Addgene #22036) and pCAG-VSVG (gift from Arthur Nienhuis & Patrick Salmon, Addgene #35616) in a 4:2:1 ratio in serum free DMEM supplemented with 3x w/w excess of polyethyleneimine 25K (Alfa Aesar, 43896). After 16 h and 24 h, medium of the LentiX cells was changed to supplemented DMEM. 40-45 h after transfection, the viral supernatant was collected and filtered through a 0.45 µm filter. Viral supernatant and 4 µg/ml polybrene (Sigma-Aldrich, 107689) were added to the target cells at 50-60% confluency at a ratio that leads to a transduction efficiency < 30% to ensure single plasmid integration. Media was changed the next day and cells were selected with 1 µg/ml Puromycin (InvivoGen, ant-pr) for one week. Derived cell lines were subsequently transduced with ecotropically packaged retroviruses

Retroviral transduction of cells. For retroviral packaging of shRNA expressing vectors, 70-80% confluent LentiX cells in supplemented DMEM were transfected with the plasmid of interest and the two packaging vectors pCMVR8.74 and pCAG-Eco (gift from Arthur Nienhuis & Patrick Salmon, Addgene #35617) in a 4:2:1 ratio in serum free DMEM supplemented with 3x w/w excess of polyethyleneimine 25K. After 16 h and 24 h, medium of the LentiX cells was changed to

supplemented DMEM. 40-45 h after transfection, the viral supernatant was collected and filtered through a 0.45 µm filter. The viral supernatant and 4 µg/ml polybrene were added to the Tet-on competent target cells at 50-60% confluency at a ratio that leads to a transduction efficiency < 30% to ensure single plasmid integration. Media was changed the next day. For selection of shRNA expressing cells, cells were treated with 1-3 mg/ml G418 solution (Roche, Roche 04727894001). shRNA expression was induced by the addition of 1 µg/ml Doxycycline (Dox) (Sigma-Aldrich, D3447).

Competitive Proliferation Assay

A2780 and A2780cis cells carrying shRNA expressing plasmids were seeded into a 24-well plate at 1 x 105 cells/well and knockdown was induced by addition of 1 µg/ml Doxycycline (Dox) (Sigma-Aldrich, D3447). After 7 days of knockdown induction, the population was divided and treated with vehicle or 1 µM cPt. The percentage of shRNA-expressing (GFP-positive) cells was measured every 2-3 days using flow cytometry. Cells were analyzed for a total of 16 days. The competitive long-term Proliferation assay was performed in the same manner. Only after 7-8 days of knockdown induction all cells were treated with cPt. Where possible cells were analyzed for percentage of shRNA-expressing (GFP-positive) cells and cell count/ml every 2-3 days. However due to the low amount of cells before resistance-acquisition, cells could not always be measured every 2 -3 days. Cells were analyzed for a total of 42 days.

MBD3 protein expression western blot

Retrovirally transduced A2780 cells carrying shRNA expressing plasmids were selected using 3 mg/ml G418-solution (Roche, 4727894001) for 7 days. Knockdown was induced for 7 days by addition of 1 µg/ml Doxycycline. 5 x 10^6 GFP-positive cells for each shRNA were acquired by SH800S Cell Sorter (Sony), washed with PBS once then then lysed in cell lysis buffer (Cell Signaling Technology, 9803) on ice for 30 min. After 10 min of incubation, the lysate was sonicated on ice for 4 cycles of 20 seconds using and EpiShear Probe Sonicator (Active Motif) to release nuclear proteins. Lysates were then centrifuged for 5 min at 10,000 x g and 4°C and the supernatant mixed with 2X laemmli protein loading buffer (125 mM Tris-HCl pH 6.8, 5% SDS, 0.004% Bromophenol Blue, 10% β-mercaptoethanol, 20% glycerol) supplemented with 100 mM DTT. Samples were then incubated at 95°C for 10 min. Proteins were resolved by SDS PAGE on a 12% polyacrylamide gel and subsequently transferred to an Immobilon-FL PVDF membrane at 300 mA for 90 min using a wet-tank blotting system (BioRad). Unspecific antibody binding sites were blocked by incubating the membrane in 5% BSA in TBST (100 mM Tris, 150 mM NaCl, 0.1% Tween) for 2 h at room temperature. Membranes were then stained with primary antibodies anti-MBD3 (1:5000), anti-Histone H3 (1:2000) or anti-β-actin (1:5000) diluted in in 5% BSA in TBST over night at 4°C. Then membranes were washed with TBST four time for 10 min and then stained with secondary antibodies IRDye® 800CW Donkey anti-Rabbit IgG (1:15000) or IRDye® 680RD

Donkey anti-Mouse IgG (1:15000) diluted in 5% BSA in TBST for 2h at room temperature in the dark. Following secondary antibody incubation, membranes were washed four times with TBST for 5 min and then imaged using the Odyssey Infrared Image System (LI-COR Biosciences GmbH).

Multiplexed RNAi screen

After spiking in several control shRNAs at equimolar ratio into an shRNAmir library targeting 1139 chromatin associated genes via 6485 shRNAs, the library was transduced in triplicates into A2780 cells. In order to ensure a 1000X library representation in the cells, 80 Mio cells were infected with a transduction efficiency of 10% to ensure only single retroviral integrations of the library. Cells were selected with 1 mg/ml G418-solution for 7 days, during which time more than 8 Mio cells were maintained at each passage to preserve library representation. After 7 days of selection, the replicates were divided and treated with 1 mg/ml G418-solution and vehicle or 1 µM cPt for an additional 7 days. During this time, cells were passaged every 2-3 days and a total amount of 8 Mio cells maintained at each passage to preserve library representation. Subsequently, 8 Mio GFP-positive cells for each replicate and treatment condition were acquired by SH800S Cell Sorter (Sony). Genomic DNA was extracted from the cells by two rounds of phenol extraction using PhaseLock tubes followed by isopropanol precipitation. Deep-sequencing libraries were generated by PCR amplification of shRNA guide strands using primers that tag the product with standard Illumina adapters (p71Loop: CAAGCAGAAGACGGCATACGATAGTGAAGC CACAGATGT; p51PGK: AATGATACGGCGACCACCGATGGATGTGGAAT GTGTGCGAGG). For each sample, DNA from at least 8 mio cells was used in multiple parallel 50-µL PCR reactions, each containing 1 µg template, 1X AmpliTaq Gold buffer, 0.2 mM of each dNTP, 2 mM $MgCl_2$, 0.3 µM of each primer and 1.25 U AmpliTaq Gold Polymerase (Invitrogen, 4311820). PCR was run using the following cycling parameters: 95°C for 10 min; 35 cycles of {95°C for 30s, 52°C for 45 s and 72°C for 60 s}; 72°C for 7min. Products of the first PCR (340 bp) were combined for each sample, column purified using the QIAquick PCR purification kit (Qiagen) and further purified on a 1% agarose gel (QIAquick gel extraction kit, Qiagen). Libraries were analyzed on an Illumina HiSeq 3000 deep sequencer; 22 nucleotides of the guide strand were sequenced using a custom primer (miR30EcoRISeq, TAGCCCCTT GAATTCCGAGGCAGTAGGCA). We acquired at least 150 average reads for each shRNA in the pools to provide a sufficient baseline for detecting shRNA depletion and to compensate for variation in shRNA representation inherent in the pooled plasmid preparation or introduced by PCR biases. Sequencing data was analyzed using the galaxy platform (https://usegalaxy.eu/) (2). For each shRNA and condition, the number of matching reads was normalized to the total number of library-specific reads per lane and imported into Microsoft Excel for further analysis. All primary screen data are provided under Supplementary Table S8. Depletion of shRNAs was determined by calculating the geometric

mean of each shRNA in each sample and dividing it by the geometric mean of the shRNAs in the pool. The gene score was determined by the sum of the depletion of all shRNAs per gene.

Immunofluorescence staining

Retrovirally transduced A2780 cells carrying shRNA expressing plasmids were selected using 3 mg/ml G418-solution for 7 days. Knockdown was induced by addition of 1 µg/ml Doxycycline for 7 days. 2 x 105 cells were seeded onto Poly-L-Lysin (Sigma-Aldrich, P4707) coated microscopy coverslips and treated with 1 µM cPt or vehicle for 24 h. Washing steps were always performed three times for 5 min with PBS$^{Mg2+\ Ca2+}$ (Sigma-Aldrich, D8662). Cells were washed and then fixed with 1 ml 4% Paraformaldehyde (Thermo Fisher Scientific, 28908) in PBS for 10 min at room temperature. Cells were washed with PBS and then permeabilized with 1 ml ice cold 0.2% Triton X-100 (Sigma-Aldrich, X100) in PBS$^{Mg2+\ Ca2+}$ for 5 min at 4°C. Cells were washed again with PBS and then incubated shaking in blocking solution (5% (w/v) blotting grade milk powder in PBS$^{Mg2+\ Ca2+}$) for 1 h at room temperature. Following blocking the cells were stained with primary antibody anti-γH2A.X (1:500) or anti-Rad51 (1:1000) diluted in 5% blotting grade milk powder in PBS$^{Mg2+\ Ca2+}$ at 4°C overnight. Cells were then washed with PBS and subsequently stained with secondary antibody Alexa Fluor 594 diluted 1:1000 in 5% blotting grade milk powder in PBS for 2 h at room temperature in the dark. Cells were washed again with PBS and then cell nuclei stained with 1 µg/ml of DAPI (Sigma-Aldrich, D9542) 1:5000 in PBS$^{Mg2+\ Ca2+}$ for 3 min at room temperature in the dark. Cells were washed again with PBS and then mounted onto a microscopy slide using Mowiol® 4-88 (Sigma-Aldrich, 81381).

Image acquisition and analysis

Images were acquired using a Zeiss Cell Observer Z1 epifluorescence microscope equipped with an Axiocam 503 mono CCD camera, and a Plan-Apochromat 63x/1.4 Oil DIC M27 objective. All channels were acquired using a colibri LED light source for excitation (blue channel: 365 nm LED, green channel: 470 nm LED, red channel: 555 nm LED) in combination with a triple bandpass filter set (Zeiss Filterset 62 HE, Colibri). Images of the whole nucleus were taken by acquisition of a Z-stack consisting of 10 images covering a total depth of 4.5 µm. Image processing was performed with ZEN blue version 2.3 (Zeiss): Hereby, images were subjected to deconvolution using a constrained iterative algorithm before generating maximum intensity projections. Quantitative analysis of speckles in each nucleus of shRNA expressing cells was performed by CellProfiler version 3.1.8 (3). In brief, nuclei were determined by DAPI staining, shRNA expressing cells as cells with a mean GFP intensity ≥ 0.04.

Gene expression analysis

To analyze mRNA expression levels, A2780 cells were treated with 1 µM cPt for 0 h, 72 h, 96 h or until they started to proliferate again under prolonged treatment with 1 µM cPt, suggesting that

they gained resistance to cPt. These newly resistant cells were harvest 26 or 42 days post resistance development. 2.5 x 10^6 cells were harvested and mRNA extracted using the RNeasy Plus Mini Kit (QIAGEN, 74134). mRNA was transcribed into cDNA using MultiScribe™ Reverse Transcriptase (Thermo Fisher Scientific, 4311235). Gene expression was then determined by real time quantitative PCR (RT-qPCR) using the CFX Real-Time PCR detection system (Bio-Rad). SDHA expression was used for normalization. Primers used for RT-qPCR are described in Supplementary Table S7.

ChIP-qPCR

For ChIP-qPCR, A2780 cells were treated with 1 µM cPt for 0 h, 72 h or until they started to proliferate again under prolonged treatment with 1 µM cPt, suggesting that they gained resistance to cPt. These newly resistant cells were harvest 26 days post resistance development. 2.5 x 10^6 cells were harvested, centrifuges at 300 x g for 5 min and pellet washed with PBS supplemented with 500 nM Trichostatin A (Sigma-Aldrich, T1952). Cells were lysed in 125 µl lysis buffer (10 mM Tris-HCl pH 7.4, 2 mM $MgCl_2$, 0.6% Igepal-Nonidet P40, 0.5 mM PMSF, 1 mM DTT, cOmplete™ EDTA-free PIC, 5 mM sodium-butyrate) for 15 min on ice. Samples were then digested using 300 U of micrococcal nuclease for 15 min at 37°C. After 15 min the samples were put on ice and the reaction stopped by addition of 8 µM EDTA, 0.1% Triton X-100 and 0.1% sodium deoxycholate solution. Samples were vortexed for 30 s and then diluted with 800 µl Complete IP buffer (20 mM Tris-HCl pH 8.0, 2 mM EDTA, 150 mM NaCl, 0.1% Triton X-100, 1 mM PMSF, cOmplete™ EDTA-free PIC, 5 mM sodium-butyrate). Mononucleosomes isolated from *Drosophila melanogaster* were added to the chromatin samples as spike-in control (5% of total chromatin). Samples were incubated for 1 h at 4°C and then centrifuged at 13,000 x g for 10 min at 4°C to pellet remaining cellular debris. 25 µl of Dynabeads™ Protein G (Thermo Fisher Scientific, 10004D) per sample were washed with Complete IP buffer three times and then resuspend in 200 µl Complete IP buffer and 2.5 µg of H3K27ac or rabbit IgG antibody. Bead were incubated with the antibodies for 4 h at 4°C at constant rotation to allow the formation of antibody bead complexes. At the same time, 50 µg of isolated chromatin was pre-cleared by incubation with 25 µl Dynabeads™ Protein G (Thermo Fisher Scientific, 10004D) and 4 µg rabbit IgG for 3-4 h at 4°C and constant rotation. Beads were removed from the chromatin samples using a magnetic rack and the pre-cleared chromatin samples were divided in half for ChIP/IgG samples. 10% of each sample was saved as input and the samples were then added to the respective antibody-bead-complexes. Chromatin and beads were incubated over night at 4°C with rotation. The next day, unbound chromatin in the supernatant was discarded and the beads with bound chromatin were washed twice with low salt wash buffer (20 mM Tris-HCl pH 8.0, 2 mM EDTA, 150 mM NaCl, 1% Triton X-100, 0.1% SDS, 5 mM sodium-butyrate) and twice with high salt wash buffer (20 mM Tris-HCl pH 8.0, 2 mM EDTA, 500 mM NaCl, 1% Triton X-100, 0.1% SDS, 5 mM sodium-butyrate) for 5 min at 4°C. Subsequently, 200 µl of ChIP elution buffer (100 mM NaHCO3, 1%

SDS) were added to the beads and the saved input samples together with 2.4 U (60 μg) Proteinase K (NEB, P8107S) and incubated for 2 h at 65°C with constant shaking. DNA in the supernatant was then purified using the ChIP DNA Purification Kit (Active Motif, 58002). Samples were quantified using the CFX Real-Time PCR detection system (Bio-Rad) and normalized to the input and the Drosophila spike-in control. Primers used for ChIP-qPCR are described in Supplementary Table S8.

ChIP-seq

For ChIP-seq of sensitive and resistant cells, 2.5 x 10^6 cells were harvested, centrifuges at 300 x g for 5 min and pellet washed with PBS supplemented with 500 nM Trichostatin A. For ChIP-seq of knockdown A2780 cells, retrovirally transduced A2780 cells carrying shRNA expressing plasmids were selected using 3 mg/ml G418-solution for 7 days. Knockdown was induced for 7 days by addition of 1 μg/ml Doxycycline. 5 x 10^6 GFP-positive cells for each shRNA were acquired by SH800S Cell Sorter (Sony), centrifuges at 300 x g for 5 min and pellet washed with PBS supplemented with 500 nM Trichostatin A. Cells were lysed in 50 μl lysis buffer (10 mM Tris-HCl pH 7.4, 2 mM MgCl2, 0.6% Igepal-Nonidet P40, 0.5 mM PMSF, 1 mM DTT, cOmplete™ EDTA-free PIC) for 15 min on ice. Samples were then digested using 120 U of micrococcal nuclease for 15 min at 37°C. After 15 min the samples were put on ice and the reaction stopped by addition of 10 μM EDTA, 0.1% Triton X-100 and 0.1% sodium deoxycholate solution. Samples were incubated on ice for 15 min, vortexed for 30 s and then diluted with 800 μl Complete IP buffer (20 mM Tris-HCl pH 8.0, 2 mM EDTA, 150 mM NaCl, 0.1% Triton X-100, 1 mM PMSF, cOmplete™ EDTA-free PIC). Samples were incubated for 1 h at 4°C and then centrifuged at 13,000 x g for 10 min at 4°C to pellet remaining cellular debris. 25 μg of chromatin for each sample were prepared and mononucleosomes isolated from Drosophila melanogaster were added to the chromatin samples as spike-in control (5% of total chromatin). 20 μl of Dynabeads™ Protein G (Thermo Fisher Scientific, 10004D) per sample were washed with Complete IP buffer three times and then resuspend in 200 μl complete IP buffer and 2.5 μg of H3K27ac, H3K9ac, mouse IgG or rabbit IgG antibody respectively. Bead were incubated with the antibodies for 4 h at 4°C at constant rotation to allow the formation of antibody bead complexes. Chromatin samples was pre-cleared by incubation with 20 μl Dynabeads™ Protein G (Thermo Fisher Scientific, 10004D) and 2.5 μg mouse or rabbit IgG for 4 h at 4°C and constant rotation. Beads were removed from the chromatin samples using a magnetic rack and 10% of each sample was saved as input. Pre-cleared chromatin was then added to the antibody-bead-complexes. Chromatin and beads were incubated over night at 4°C with rotation. The next day, unbound chromatin in the supernatant was discarded and the beads with bound chromatin were washed twice with low salt wash buffer (20 mM Tris-HCl pH 8.0, 2 mM EDTA, 150 mM NaCl, 1% Triton X-100, 0.1% SDS) and twice with high salt wash buffer (20 mM Tris-HCl pH 8.0, 2 mM EDTA, 500 mM NaCl, 1% Triton X-100, 0.1% SDS) for 10 min at 4°C. Subsequently, 200 μl of ChIP elution buffer (100 mM NaHCO3, 1% SDS) was

added to the beads and the saved input samples together with 2.4 U (60 μg) Proteinase K and incubated for 2 h at 65°C with constant shaking. DNA in the supernatant was then purified using the ChIP DNA Purification Kit. ChIP libraries were prepared and sequenced by the Max Planck Genome center Cologne. Sequencing was performed on an Illumina HiSeq 3000 deep sequencer acquiring 2 x 150 bp paired end reads aiming at 10 mio total reads.

ChIP-Seq data analysis

Sequencing data was analyzed using the galaxy platform (https://usegalaxy.eu/) (2). Paired-end reads were aligned to the human hg19 genome using Bowtie2 (Galaxy Version 2.3.4.3) (4). The ChIP-seq data was then binned into genomic intervals of 200 bp using BedCov (Galaxy Version 2.0.2) and subsequently quantile normalized using an R script employing the quantile normalization function of the R package 'preprocessCore' (5) or a custom python script. Both scripts are available on GitHub (https://github.com/Rathert-lab**).** Input samples and IP samples were always normalized separately. Quantile normalized bedgraph files were converted into bigwig files using Wig/BedGraph-to-bigWig converter (Galaxy Version 1.1.1). Peaks in the ChIP-seq data were identified using the MACS2 peak calling algorithm (5) and the MACS2 package on galaxy. In a first step, noise reduction was performed by comparing the input (control) samples to the IP samples using MACS2 bdgcmp (Galaxy Version 2.1.1.20160309.0). Broad peaks were then called from the bedGraph datasets using MACS2 bdgbroadcall (Galaxy Version 2.1.1.20160309.0). 1500 was used as a cutoff for peaks, 750 as a cutoff for linking regions for all A2780 and A2780cis ChIP-seq data. For CAOV4/CAOV4cis ChIP-seq data, which overall had much lower peaks, the cutoff for peaks was adapted to 1000 and the cutoff for linking peaks to 500. The resulting genomic interval files were then converted into bed files using the tool Convert Genomic Intervals To BED (Galaxy Version 1.0.0).

Visualization and identification of differential peaks. ChIP-seq tracks were visualized using the Integrative Genomics Viewer version 2.6.3 (6). For generation of heatmaps, generated Bigwig and bed files were loaded into ChAsE version 1.1.2 (chromatin analysis and exploration tool) (7). All regions were resized to 10,000 bp with 200 bp bins. Differential regions were identified by the ChAsE inbuilt K-means clustering algorithm. For differential peak filtering, peaks for all ChIP-seq data of interest were concatenated in Galaxy using Concatenate datasets (Galaxy Version 1.0.0). Overlapping intervals of the dataset were then merged using Merge (Galaxy Version 1.0.0) and subsequently clustered using Cluster (Galaxy Version 1.0.0). Average ChIP-seq signal for each of these clustered regions in the different condition was determined using the multiBigwigSummary function (Galaxy Version 3.0.2.0) of the deepTools2 tool set (8). Differential peak filtered was then performed on the average signal for each region by filtering for peaks with a locus length > 2 kb, an average peak intensity of > 1000 and a fold change between upregulated and downregulated regions of > 1.3 or < 0.75 respectively.

Correlation analysis. For correlation analysis, average ChIP-seq signal in each of the regions used for correlation analysis was calculated using multiBigwigSummary. Spearman correlation coefficients were then determined using the plotCorrelation function (Galaxy Version 3.0.2.0) of deepTools2 (8).
Identification of super-enhancers. Super-enhancers were called using the Rank Ordering of Super-Enhancers (ROSE) tool (9). ROSE was run on the BwUniCluster 2.0 using Python version 2.7.12, Samtools version 1.9 and R version 3.3.1.

Supplementary References

1. Scholz C, Heublein S, Lenhard M, Friese K, Mayr D, Jeschke U. Glycodelin A is a prognostic marker to predict poor outcome in advanced stage ovarian cancer patients. BMC Res Notes. 2012;5:551.
2. Afgan E, Baker D, Batut B, Van Den Beek M, Bouvier D, Ech M, Chilton J, Clements D, Coraor N, Grüning BA, et al. The Galaxy platform for accessible, reproducible and collaborative biomedical analyses: 2018 update. Nucleic Acids Res. 2018;46:W537–44.
3. McQuin C, Goodman A, Chernyshev V, Kamentsky L, Cimini BA, Karhohs KW, Doan M, Ding L, Rafelski SM, Thirstrup D, et al. CellProfiler 3.0: Next-generation image processing for biology. Misteli T, editor. PLOS Biol. 2018;16:e2005970.
4. Langmead B, Salzberg SL. Fast gapped-read alignment with Bowtie 2. Nat Methods. 2012;9:357–9.
5. Feng J, Liu T, Qin B, Zhang Y, Liu XS. Identifying ChIP-seq enrichment using MACS. Nat Protoc. 2012;7:1728–40.
6. Robinson JT, Thorvaldsdóttir H, Winckler W, Guttman M, Lander ES, Getz G, Mesirov JP. Integrative genomics viewer. Nat. Biotechnol. 2011. page 24–6.
7. Younesy H, Nielsen CB, Lorincz MC, Jones SJM, Karimi MM, Möller T. ChAsE: Chromatin analysis and exploration tool. Bioinformatics. 2016;32:3324–6.
8. Ramírez F, Ryan DP, Grüning B, Bhardwaj V, Kilpert F, Richter AS, Heyne S, Dündar F, Manke T. deepTools2: a next generation web server for deep-sequencing data analysis. Nucleic Acids Res. 2016;44:W160–5.
9. Whyte WA, Orlando DA, Hnisz D, Abraham BJ, Lin CY, Kagey MH, Rahl PB, Lee TI, Young RA. Master transcription factors and mediator establish super-enhancers at key cell identity genes. Cell. 2013;153:307–19.
